# In-depth characterization of phytase-producing plant growth promotion bacteria isolated in alpine grassland of Qinghai-Tibetan Plateau

**DOI:** 10.3389/fmicb.2022.1019383

**Published:** 2023-01-04

**Authors:** Qi Li, Xiaolei Yang, Jianhong Li, Mingyuan Li, Changning Li, Tuo Yao

**Affiliations:** Key Laboratory of Grassland Ecosystem, Ministry of Education, Sino-U.S. Centers for Grazing Land Ecosystem Sustainability, Ministry of Science and Technology, College of Pratacultural Science, Gansu Agricultural University, Lanzhou, China

**Keywords:** phytase, plant growth-promoting bacteria, plant growth promotion, β-propeller phytase, *Pseudomonas mandelii* GS10-1, heterologous expression

## Abstract

The use of plant growth promoting bacteria (PGPB) express phytase (*myo*-inositol hexakisphosphate phosphohydrolase) capable of hydrolyzing inositol phosphate in soil was a sustainable approach to supply available phosphorus (P) to plants. A total of 73 bacterial isolates with extracellular phytase activity were selected from seven dominant grass species rhizosphere in alpine grassland of Qinghai-Tibetan Plateau. Then, the plant growth promoting (PGP) traits of candidate bacteria were screened by qualitative and quantitative methods, including organic/inorganic Phosphorus solubilization (P. solubilization), plant hormones (PHs) production, nitrogen fixation, 1-aminocyclopropane-1-carboxylic acid (ACC) deaminase activity and antimicrobial activity. Further experiment were conducted to test their growth promoting effect on Lolium perenne L. under P-limitation. Our results indicated that these bacteria as members of phyla Proteobacteria (90.41%) and Actinobacteria (9.59%) were related to 16 different genera. The isolates of *Pseudomonas* species showed the highest isolates number (36) and average values of phytase activity (0.267 ± 0.012 U mL^–1^), and showed a multiple of PGP traits, which was a great candidate for PGPBs. In addition, six strains were positive in phytase gene (β-propeller phytase, *bpp*) amplification, which significantly increased the shoot length, shoot/root fresh weight, root average diameter and root system phytase activity of *Lolium perenne* L. under P-limitation, and the expression of phytase gene (*bppP*) in root system were verified by qPCR. Finally, the PHY101 gene encoding phytase from *Pseudomonas mandelii* GS10-1 was cloned, sequenced, and recombinantly expressed in *Escherichia coli*. Biochemical characterization demonstrated that the recombinant phytase PHY101 revealed the highest activity at pH 6 and 40°C temperature. In particular, more than 60% of activity was retained at a low temperature of 15°C. This study demonstrates the opportunity for commercialization of the phytase-producing PGPB to developing localized microbial inoculants and engineering rhizobacteria for sustainable use in alpine grasslands.

## Introduction

Over the past few decades, to prevent food shortage worldwide, meat and milk consumption have increased markedly. In particular, the grasslands are important contributors to global food security by supplying proteins and energy to ruminants (Sattari et al., 2016; Stromberg and Staver, 2022). However, grassland degradation has accelerated given the accelerated growth of animal husbandry, soil nutrient loss from overgrazing, weed encroachment, and desertification (Granada et al., 2018). Sustainably meeting forage grass demand is one of the grand challenges of agricultural production, and phosphorus (P) is increasingly found to be a new global sustainability issue given the increasing depletion of extractable P rocks (Dhillon et al., 2017; Granada et al., 2018). Similar to nitrogen, P is an essential nutrient in agriculture and is removed from grassland soils by grass withdrawal and erosion (Sattari et al., 2016). The P removed during the harvest of grass needs to be replaced by inputs through organic and mineral fertilizers to sustain grass production.

Phytate (*myo*-inositol hexakisdihydrogen phosphate, IHP) is a class of inositol phosphate found widely in the natural environment (Turner et al., 2002). They are synthesized in terrestrial ecosystems by plants and strongly complexed in soils, where they accumulate to form the dominant component of organic phosphorus (P_o_) (Menezes-Blackburn et al., 2013). The survey of global P storage suggested that the potential of the use of monoester P (mostly inositol phosphates) (George et al., 2018) is greater for grasslands than for arable soils (Menezes-Blackburn et al., 2018). However, due to the lack of active substances that hydrolyze phytate, it is not easy for plants to obtain P from them (Gerke, 2015). In contrast, microorganisms have been shown to efficiently degrade it (Li et al., 2018; Ding et al., 2021; Li J. T. et al., 2021). The P deficiency can significantly enhance the ability of microbial communities to extract P from soil (Jarosch et al., 2019) and increase the abundance of phosphohydrolase-related genes, such as phytase (Yao et al., 2018; Park et al., 2022). Phytase (*myo*-inositol hexakisphosphate phosphohydrolase) produces P by hydrolyzing phytate (Lei et al., 2013). Many bacteria have been reported to secrete phytases, such as *Pseudomonas*, *Bacillus*, *Achromobacter*, *Acinetobacter*, *Klebsiella*, *Micrococcus*, *Burkholderia*, and *Serratia* (Jorquera et al., 2018; Neal et al., 2018; Wan et al., 2020; Rix et al., 2021), which are isolated in different habitats, and support its great potential as a suitable phytase producer with desired properties. Three groups of phytase encoding β-propeller phytases (BPPhy), cysteine phytases (CPhy), and histidine acid phytases (HAPhy) were found in bacteria (Singh et al., 2020), among which BPPhy was the most widely distributed and was considered a good biomarker for evaluating soil organic P transformation (Kumar et al., 2017; Lin et al., 2021). However, despite their importance in soil P mineralization, the study of phytase addressing the P cycle in grasslands soil remains poorly understood. Its diversity, bacterial communities involved in its secretion, and conditions that may influence its production and reactivity need to be elucidated (Devi et al., 2022).

Plant growth-promoting bacteria (PGPB) is a group of well-known microorganisms that can promote plant growth, which can enhance nitrogen fixation, synthesize growth regulators or plant hormones, dissolve organic/inorganic phosphates, protect against harmful effects of soil-borne plant pathogens, etc. (Tapia-García et al., 2020; Hu et al., 2021; Li M. Y. et al., 2021). However, most global studies mainly focus on arable land and ignore the huge biotechnological potential of PGPBs in grasslands (Haskett et al., 2021; Pankievicz et al., 2021). Additionally, little attention has been given to the screening for PGPBs by identifying microbial-containing phosphatase encoding genes (Wan et al., 2020). The plants in combination with PGPBs possessing the ability to produce phytase and promote plant growth is an efficient and environmentally sustainable strategy used in the restoration of degraded grasslands (Granada et al., 2018) since it presents the potential to reduce the amount of the most important synthetic inputs applied on grasses, which is the paramount importance of fertilizers obtained from limited sources (Alori et al., 2017). The availability and stability of PGPBs are affected by various factors, such as soil physicochemical properties, climatic conditions, and the composition of microbial communities. Therefore, the understanding of native bacterial populations, finding region-specific microbial, and collecting and establishing a database of isolates are necessary to advance the wider commercial use of PGPBs (Goh et al., 2019; Vasseur-Coronado et al., 2021).

The Qinghai-Tibet Plateau is the largest and highest plateau on earth and also the main distribution area of alpine grasslands in China that has unique climatic characteristics, such as low temperature, hypoxia, and nutritional deficiencies, and breeds abundant microbial resources (Ma et al., 2020). Therefore, PGPBs living there potentially represent new species and develop unique physiological adaptation mechanisms, ultimately producing bioactive compounds. The aims of the present study are as follows: (1) using the culture-dependent approach to investigate the cultivable phytase-producing bacterial diversity in the rhizosphere; (2) assessing the PGP traits of isolates quantitatively under suitable conditions; (3) comparing the effect of inoculation of strains on plant growth under P-limitation; and (4) cloning, sequencing, and expressing the phytase genes from strains, and evaluating its biochemical properties. We anticipate that several bacteria with phytase activity and PGP traits will be obtained in this study, and the new phytase with activities will be revealed clearly.

## Materials and methods

### Sample collection

The samples were collected from seven dominant types of grass, including *Astragalus chilienshanensis*, *Avena sativa*, *Bupleurum chinense*, *Kobresia kansuensis*, *Oxytropis ochrocephala*, *Poa pratensis*, and *Stipa capillata* from the Menyuan County, Qinghai Province, China (37°39′51″N, 101°10′44″W) in August 2019, which is located in the northeast side of the Qinghai-Tibet Plateau. The healthy individual plants were collected by using sterile tools, and the whole roots were carefully dug out and shaken to remove the bulk soil while retaining the tightly bound soil. Samples were kept in sterile plastic bags, transported to the laboratory on ice, and used immediately for the isolation of strains of bacterial cultures.

### Isolation and screening of phytase-producing bacteria

The isolates were screened from samples using the dilution-plate method in modified medium M1 containing sucrose 10 g, (NH_4_)_2_SO_4_ 0.5 g, NaCl 0.3 g, KCl 0.3 g, MnSO_4_⋅4H_2_O 0.03 g, FeSO_4_⋅7H_2_O 0.03 g, yeast extract 0.4 g, calcium phytate 5 g, and agar 18 g and medium M2 containing glucose 10 g, (NH_4_)_2_SO_4_ 0.1 g, MgCl_2_⋅6H_2_O 5 g, MgSO_4_⋅7H_2_O 0.25 g, KCl 0.2 g, calcium phytate 5 g, and agar 18 g (Nautiyal, 1999). Approximately 1 g of fresh root samples was added to 9 ml of 0.9% normal saline and serially diluted to 10^–3^, 10^–4^, and 10^–5^ dilutions. Then, 100 μl of diluted samples were spread-plated on the medium and incubated at 28°C for 4–6 days. The strains were selected by their color morphology and surface smoothness, purified two to three times to obtain pure cultures, and cryopreserved in 20% glycerol at –80°C.

The isolates were inoculated in Luria-Bertani (LB) liquid medium for 24 h. The cultures were centrifuged and suspended in sterile water until the optical density (OD) reached 1.0 at 600 nm. The cell suspension was inoculated in phytase-screening medium containing glucose 20 g, NH_4_NO_3_ 2 g, KCl 0.5 g, K_2_HPO_4_ 0.04 g, MgSO_4_⋅7H_2_O 0.5 g, MnSO_4_⋅4H_2_O 0.01 g, FeSO_4_⋅7H_2_O 0.01 g, and CaCl_2_⋅2H_2_O 0.04 g at pH 7.0 and incubated at 15°C, 200 rpm for 5 days. Then, 10 ml of each culture was centrifuged at 12,000 × *g* for 15 min and the supernatant was used to assay extracellular phytase activity.

Phytase activity was measured by the ferrous sulfate-molybdenum blue colorimetry method (Bae et al., 1999). Under standard conditions, the reaction mixture contained 0.1 ml of culture supernatant, 2 mM CaCl_2_, 1.5 mM sodium phytate (Sigma-Aldrich P3168, DE, USA), and 0.1 M Tris–HCL buffer (pH 7.0) in order to bring the final volume to 1 ml. The reaction mixture was incubated for 30 min at 37°C and the reaction was terminated by the addition of 1 ml 10% (w/v) trichloroacetic acid (TCA). Then, 2 ml of color reagent containing 1% (w/v) ammonium molybdate, 3.2% (v/v) sulfuric acid, and 7.32% (w/v) ferrous sulfate was added. The released inorganic phosphate was analyzed by standing at room temperature for 10 min followed by centrifugation (13,000 × *g*, 4°C) for 10 min. One unit of phytase activity was defined as the amount of enzyme catalyzing the release of 1 μmol of P per min under the above-specified conditions. Each experiment was replicated three times.

### 16S rRNA genes amplification and phylogenetic analysis

The genomic DNA of isolates was extracted using Bacteria Genomic DNA Extraction Kit (Takara No. 9763, Beijing, China). The 16S rRNA gene of strains was amplified using the universal primer 27F-1492R following the PCR conditions described by Frank et al. (2008). Both genomic DNA and PCR product quality and quantity were determined in NanoPro (2010) (DHS Life Science and Technology, Beijing, China). Samples were sent to Shanghai Sangon Biological Engineering Co., Ltd. (Shanghai, China) for sequencing using the Sanger method. The sequences of strains of the 16S rRNA gene were identified, and the most similar strains were downloaded by Ezbiocloud^1^ (Yoon et al., 2017). Then, the phylogenetic tree of the 16S rRNA gene was aligned by ClustalX and constructed by the Neighbor-joining method (Saitou and Nei, 1987) with bootstrap based on 1,000 replications in MEGA 7.0, and the nucleotide sequences of the strains 16S rRNA gene fragments were deposited into GenBank under the accession numbers OP412497-OP412569.

### Determination of *in vitro* PGP traits

The isolates belonging to *Pseudomonas* species (*n* = 36) were selected to determine *in vitro* plant growth-promoting (PGP) traits.

#### Phosphorus solubilization

The isolates were grown in Pikovskaya’s and Mongina’s liquid medium (Li M. Y. et al., 2021) at 15°C, 180 rpm for 10 days, and centrifuged at 10,000 × *g* for 20 min to obtain the supernatant. The inorganic (tricalcium phosphate) and organic (egg-yolk lecithin) P solubilization activities were quantified using phospho-molybdate blue color method (Nautiyal, 1999) at 700 nm. Each experiment was replicated three times.

#### Plant hormones production

The isolates were grown in LB liquid medium at 15°C, 180 rpm for 5 days in dark conditions. The production of plant hormones (PHs) was quantified by high-performance liquid chromatography (HPLC). The supernatant was extracted using ethyl acetate and analyzed on an HPLC system (Agilent Technologies 1260 Infinity, CA, USA) using Perrig et al. (2007) methods. The chromatographic column was Agilent ZORBAX Eclipse Plus C18 Column (250 × 4.6 mm, 5 μm) and the mobile phase was 45:55:1 (v:v:v), which included methanol, ultrapure water, and acetic acid in 254 nm. The standard PHs for testing, namely, trans-Zeatin (t-Z), gibberellin acid (GA_3_), and indoleacetic-3-acid (IAA), were purchased from Merck (Darmstadt, Germany), and the chromatographic peak times were 3.788 min, 5.382 min, and 10.076 min, respectively. Each experiment was replicated three times.

#### ACC deaminase activity

The isolates were suspended in 7.5 ml DF salts minimal medium, and 45 μl sterile 0.5 M 1-aminocyclopropane-1-carboxylic acid (ACC) solution was added to the culture at 15°C, 200 rpm for 24 h to induce ACC deaminase production, and the activity of isolates was determined using 2, the 4-dinitrophenylhydrazine colorimetric method by Penrose and Glick (2003) described, and the protein concentration was determined by the method of Bradford (Spector, 1978). Each experiment was replicated three times.

#### Nitrogen fixation

The isolates were inoculated into malate Ashby’s nitrogen-free medium semi-solid medium vials at 15°C for 48 h. Nitrogen fixation activity was calculated based on the results of the acetylene reduction assay (ARA) (Hardy et al., 1968). Acetylene (5% v/v) was injected into the vials and incubated at 15°C for 24 h, 50 μl of gas samples from the vials were analyzed on a gas chromatography (GC) system (Aglient Technologies 7890B, CA, USA), and the protein concentration determination method is the same as mentioned previously. Each experiment was replicated three times.

#### Antimicrobial activity

The antimicrobial activity of isolates against two plant pathogenic fungi (*Rhizoctonia solani* and *Fusarium oxysporum*) was estimated by the plate confrontation method (Li et al., 2020) in potato dextrose agar (PDA) medium. The growth inhibition ratio (%) was calculated by *I* = (D-d)/D × 100%, where “D” and “d” shows the colony diameters of the pathogen in the control and the treatment groups, respectively. Each experiment was replicated three times.

### Detection of phytase gene using PCR amplifications

The degenerate PCR methods used to detect the presence of the phytase gene included β-propeller phytase (*bpp*) (Huang et al., 2009), histidine acid phytase (*hap*) (Huang et al., 2011), and cysteine phytase (*cp*) (Huang et al., 2006). The primers’ information is shown in Supplementary Table 2. The touchdown PCR conditions are as follows: 95°C for 4 min, then 10 (*bpp*)/8 (*hap*, *cp*) cycles of 94°C for 30 s, 50°C (*bpp*)/58°C (*hap*, *cp*) for 30 s (decreasing 0.5°C of *bpp* and 1°C of *hap* and *cp* for each cycle), and 72°C for 30 s, followed by 25 cycles of 95°C for 30 s, 50°C for 30 s, and 72°C for 30 s, and a final extension step at 72°C for 10 min, 4°C hold. The 50-μl PCR reaction systems contained 25 μl Premix (1.25 U of DNA polymerase, 0.4 mM each of the dNTPs, 4 mM Mg^2+^), 0.05 mM of each primer, and 50–100 ng of bacterial genomic DNA, and verified by agarose gel electrophoresis (1.5%) using gel imager (Bio-Rad GelDoc XR, Hercules, CA, USA). The amplified fragments with appropriate size (160–200 bp) were cut by rubber recycling and ligated into the pTOPO-TA vector (Aidlab CV14, Beijing, China). The positive clones were sent to Sangon Bio for sequencing, and the results were searched using BLSATn in NCBI.^2^

### PGP potential of strains under P-limitation

Six strains containing the phytase gene were selected to test their PGP potential under P limitation. *Lolium perenne* L. “227” (Grass Germplasm Innovation Laboratory, Gansu Agricultural University, China) seeds were washed, surface-sterilized for 15 min in 2% (v/v) sodium hypochlorite, washed four times with sterile water, and the floating seeds were discarded (Ramakrishna et al., 1991). The sterilized seeds were sown in the germination box and germinated in the dark at 15°C for 4 days. Then, the seedlings were transplanted into the hydroponics unit and incubated in a biotron (Eshengtaihe Ctrl Tech, Beijing, China) under controlled conditions (15°C day/night, 18 h light/6 h dark, light intensity 1,250 μmol m^–2^ s^–1^) at Pratacultural College, Gansu Agricultural University, in each device in five replicates. Half-strength P-free Hoagland sterile nutrient solution and 100 mg L^–1^ sodium phytate were added to each device. The bacterial suspension with an OD (600 nm) value of 1.0 was added to the device after the seedlings were grown for 2 days. The Hoagland’s was changed every 7 days. The plants were harvested on day 28, and the following indicators were measured: shoot length, root length, fresh weight shoot, fresh weight root, dry weight shoot, dry weight root, and root/shoot ratio. The root morphology was obtained by using Expression 12000XL (Epson, Suwa, Japan). Then, different treatments of roots with plants were collected to determine the phytase activity and used for quantitative PCR (qPCR) detection.

The RNA of the root sample was extracted using RNAprep Pure Bacteria Kit (TIANGEN DP430 Beijing, China), and the cDNA was synthesized using a reverse transcription kit (PrimeScript RT reagent Kit with gDNA Eraser, TaKaRa No. RR047A, Beijing, China). The TB Green Premix Ex Taq™ II (TaKaRa No. RR820Q, Beijing, China) on the LightCycler 96 PCR System (Roche, Basel, Switzerland) was used to perform the qPCR procedure. The primer *bpp*P (Supplementary Table 2) was employed to evaluate the expression of strains *bpp* gene in the root system. The abundance of the 16S rRNA gene was used as inner control to calculate the relative expression. The PCR reaction conditions were referenced by Dong et al. (2020) method.

### Cloning of phytase gene

The full-length sequences of phytase gene was cloned from strain *Pseudomonas mandelii* GS10-1. The 5′ and 3′ flanking regions of the phytase gene were obtained using the Genome Walking Kit (Takara No. 6108, Beijing, China) by the manufacturer’s instructions, and the nested gene-specific primers are shown in Supplementary Table 2. The amplified PCR products were ligated into the pTOPO-TA vector for sequencing. Both upstream and downstream corrected sequences and known sequences were spliced using the Seqman program to assemble into a complete sequence, and the complete open reading frame (ORF) was predicted for the sequence using an ORF finder in the NCBI program. The similarity of the sequence encoding amino acid to other homologous sequences was compared using a Blast tool in the UniProt protein database,^3^ and the similar sequences to multiple alignments were downloaded using a ClustalX program. The SWISS-MODEL^4^ was used to perform protein structure homology modeling. The obtained phytase gene was named PHY101 (GenBank accession number: OM935859).

### Production and purification of recombinant protein

The recombinant PHY101 protein was expressed using an *Escherichia coli* expression system. The mature protein-coding gene sequence (remove signal peptide) of PHY101 and PCR *Pfu* DNA polymerase (Vazyme Biotech P525, Nanjing, China) were used for amplification. The PCR product was purified by gel extraction and sub-cloned between the *Bam*HI and *Hind*III sites of the pET-28a(+) vector. The 6 × His-tag at the N-terminal end of the target gene, and the vector named as pET-PHY101. The vector pET-PHY101 was transformed into *E. coli* BL21 competent cells by heat shock method. The recombinants were selected on LB agar medium containing kanamycin (Kana, 50 μg mL^–1^). The white colonies that appeared on plates were extracted using Plasmid Mini Kit (Omega Bio-Tek D6943, CT, USA), and PCR methods were performed for commercial sequencing identification.

The *E. coli* strain (BL21/pET-PHY101) was grown in LB liquid medium containing 50 mg L^–1^ Kana at 37°C, 220 rpm. Recombinant protein expression was induced using 1 mM IPTG until the cultures’ OD (600 nm) reached 0.8 (log phase), induction culture at 20°C for 6 h. Cells were harvested by centrifugation at 4°C and pellets were suspended in Tris–HCl (pH 7.0). The his-tagged recombinant protein PHY101 was purified using Ni-NTA Fast Start Kit (QIAGEN No. 30600, Hilden, Germany). The fraction containing the purified protein was collected for 12% sodium dodecyl sulfate polyacrylamide gel electrophoresis (SDS-PAGE). The phytase activity and protein concentration were assayed as previously described. All activity assays were performed in the presence of 5 mM Ca^2+^ unless otherwise stated.

### Enzymatic properties of recombinant phytase analysis

#### Effects of pH and temperature

The optimum pH of PHY101 was determined by measuring the relative activity of the enzyme under different pH conditions (3.0–10.0), The buffers used were 0.1 M citric acid-sodium citrate buffer at pH 3.0–5.0; 0.1 M acetic acid-sodium acetate buffer at pH 5.0–7.0; 0.1 M Tris–HCl buffer at pH 7.0–8.0; and 0.1 M glycine-sodium hydroxide buffer at pH 9.0–10.0. The stability of pH was determined by optimum pH at 37°C after the enzyme solution was treated in different pH buffers (2.0–10.0) at 37°C for 1 h.

The optimum temperature was determined by different temperature conditions (10–60°C) under optimum pH. The stability of temperature was determined by different temperatures from 10 to 60°C for 20 min, and the thermal stability was determined by optimum pH after 10–60 min for 40, 50, and 60°C.

#### Effect of metal ions and chemical reagents

The different concentrations (1 and 5 mM) of metal ions (Mg^2+^, Na^+^, K^+^, Mn^2+^, Cr^3+^, Pb^2+^, Cu^2+^, Zn^2+^, and Fe^3+^) and chemical reagents (EDTA and SDS) were added to the enzymatic reaction system to study their effects on the enzymatic activity at optimum pH and temperature, and treatment without any reagent was used as the control.

### Statistical analysis

Statistical analyses were performed using one-way analysis of variance (ANOVA) by SPSS 25.0 software. The mean comparison was carried out by using Duncan’s multiple range tests. Statistically significant data was determined at *p* ≤ 0.05. Data are presented as means ± SD.

## Results

### Phytase-producing bacteria from plant samples

**FIGURE 1:**
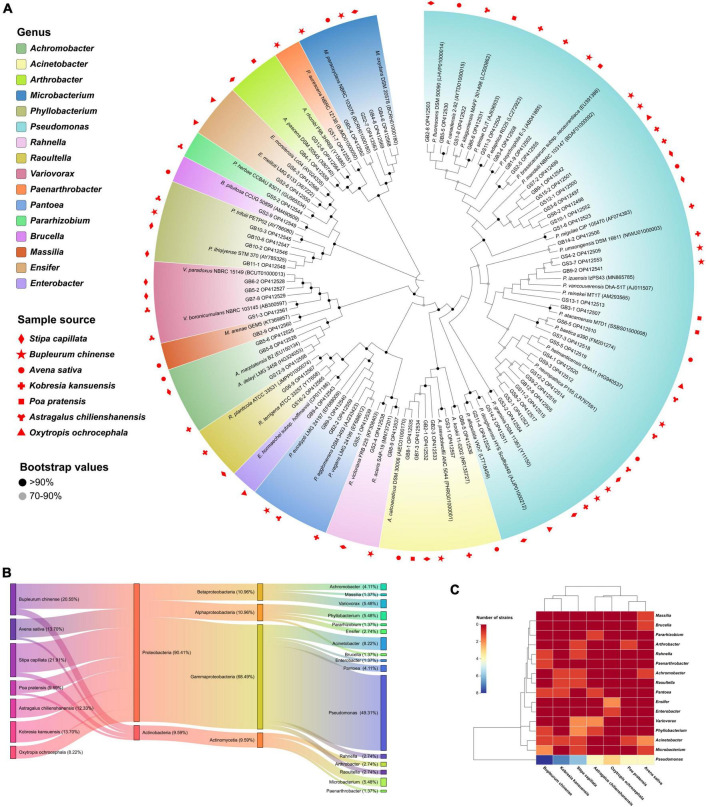
Diversity and taxonomic analysis of phytase-producing bacteria. **(A)** Neighbor-joining tree based on 16S rRNA gene sequences showing relationships between the 73 isolates and their closest relatives, the effective sequence length was 1,200 bp and the bootstrap values were 1,000 replications. The serial number in parentheses denotes the GenBank accession number of strains. **(B)** Sankey diagram of the community composition of bacteria isolates. From left to right: Host plant, phylum level, class level, and genus level. **(C)** Abundance and hierarchical clustering analysis of bacteria isolates under different plants treatment at genus level.

A total of 114 bacteria isolates were selected and purified from seven plants using enriched media, and 73 isolates (64.04%) of them have extracellular phytase activity. The 16S rRNA gene (∼1,200 bp) sequences BLAST alignment validated the identity with 99.41–100% similarity (Figure 1A). The sample details as shown in Supplementary Table 1. The cultivable phytase-producing bacteria belong to two phyla, four classes, and sixteen genera. Proteobacteria (90.41%) was the dominant phyla and the other was Actinobacteria (9.59%) (Figure 1B). *Pseudomonas* was the most abundant genus for 36 isolates (49.31%) followed by *Acinetobacter* for six isolates (8.22%). In addition, *Stipa capillata* rhizosphere yielded the highest number of isolates (16) including *Pseudomonas*, *Phyllobacterium*, *Variovorax*, *Achromobacter*, *Acinetobacter*, *Arthrobacter*, *Rahnella*, *Raoultella*, and *Microbacterium*, followed by *Bupleurum chinense* (15), and the lowest number of them was *Oxytropis ochrocephala* (6). Only *Pseudomonas* species isolates were identified in all plants tested (Figure 1C).

**FIGURE 2:**
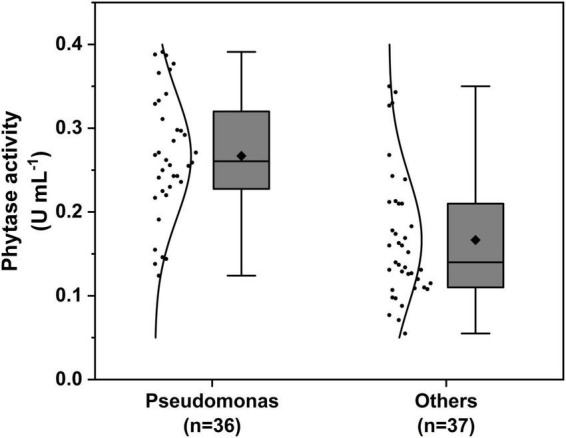
Comparison analysis of phytase activity between genera of *Pseudomonas* and others.

The phytase activity assay results of isolates are shown in Supplementary Table 1. The mean value of phytase activity of *Pseudomonas* species isolates showed a higher average of 0.267 ± 0.012 U mL^–1^, among *Pseudomonas baetica* GS6-5 from *Avena sativa* showed the highest activity (0.391 U mL^–1^), and the mean value of others genus was 0.168 ± 0.13 U mL^–1^, among *Acinetobacter calcoaceticus* GB2-3 from *Bupleurum chinense* showed the highest activity (0.350 U mL^–1^) (Figure 2).

### PGP traits of bacterial isolates

All the isolates from *Pseudomonas* (36) species were selected to evaluate different PGP traits, the antagonism of two pathogenic fungi, and the presence of three encoding phytase genes (*bpp*, *hap*, *cp*). The results showed that they had multiple PGP traits as shown in Table 1. Among these, all the isolates showed an excellent P-solubilization in the range 49.08–410.73 μg mL^–1^ ability when tricalcium phosphate was the P source, and *Pseudomonas mandelii* GS10-1 showed the highest solubilization ability, followed by *Pseudomonas mandelii* GS3-6. A total of 27 isolates (75.00%) showed P-solubilization ability in the range 0.97–27.76 μg mL^–1^when egg-yolk lecithin was the P source, *Pseudomonas baetica* GS7-3 showed the highest solubilization ability, followed by *Pseudomonas neuropathica* GS4-1.

**TABLE 1:** Screening the *in vitro* plant growth-promoting (PGP) traits in genus of *Pseudomonas.*

Strain no.	P-solubilization^a^ (μ g mL^–1^)		Plant hormones production^b^ (μ g mL^–1^)			ACCD activity^c^ (μ mol mg^–1^ protein h^–1^)	Nitrogen fixation (nmol C_2_H_4_ mg^–1^ protein h^–1^)	Antimicrobial activity^d^		PCR^e^		
	I-P	O-P	t-Z	GA_3_	IAA			*RS*	*FO*	*bpp*	*hap*	*cp*
GB1-9	49.08	0.97	-	62.22	0.11	–	51.53	–	–	–	–	–
GB2-8	247.98	-	-	53.43	0.33	–	69.93	15.77%	32.90%	–	–	–
GB3-1	85.32	8.88	8.71	29.37	0.09	–	–	–	–	–	–	–
GB3-4	179.64	10.23	4.44	41.97	1.02	–	–	–	–	–	–	–
GB5-5	99.32	1.94	6.47	56.25	-	–	85.20	–	–	+	–	–
GB6-6	234.85	9.08	-	95.67	0.14	–	66.92	12.47%	22.54%	–	–	–
GB9-1	111.41	3.15	12.36	-	-	–	80.72	–	–	–	–	–
GB9-2	271.43	2.65	-	74.98	-	–	77.66	–	–	–	–	–
GB12-9	255.54	6.13	12.01	63.39	0.20	–	90.33	24.80%	18.55%	–	–	–
GB14-2	305.55	-	-	38.55	-	0.85	69.94	–	–	–	–	–
GS1-6	309.87	6.98	11.17	-	0.17	–	88.41	6.89%	20.46%	–	–	–
GS1-8	212.63	17.45	7.50	99.40	-	–	77.07	–	–	–	–	–
GS2-1	83.95	-	9.08	-	0.32	–	–	–	–	–	–	–
GS2-5	80.90	-	-	39.62	-	–	–	–	–	–	–	–
GS3-2	278.05	4.80	16.67	67.44	1.39	–	93.20	–	–	–	–	–
GS3-6	380.01	12.37	15.91	109.51	0.87	–	–	10.37%	–	+	–	–
GS3-7	189.62	-	-	48.60	0.30	–	–	–	–	–	–	–
GS4-1	247.99	25.44	-	28.65	1.05	–	88.82	–	–	–	–	–
GS4-2	174.35	19.08	9.19	48.60	0.23	1.09	74.50	21.86%	8.60%	–	–	–
GS5-5	77.90	9.80	14.50	-	0.62	–	94.01	–	–	–	–	–
GS6-2	185.44	-	12.50	80.94	-	–	–	–	–	–	–	–
GS6-5	342.33	-	11.61	130.09	0.66	–	–	–	–	+	–	–
GS7-2	197.40	11.93	6.93	98.51	0.98	–	74.93	–	–	–	–	–
GS7-3	278.05	26.76	-	66.50	2.27	–	84.47	25.94%	20.80%	–	–	–
GS8-2	366.39	11.51	8.63	23.16	0.37	3.14	75.25	–	–	–	–	–
GS9-2	211.50	7.83	15.90	90.51	-	–	88.05	–	–	–	–	–
GS9-3	300.98	2.94	3.98	110.50	0.85	–	90.58	–	–	–	–	–
GS10-1	410.73	14.85	10.64	173.22	0.69	–	–	–	–	+	–	–
GS11-2	186.05	7.80	17.45	44.23	0.73	2.29	92.33	–	–	–	–	–
GS11-3	224.44	8.65	12.84	144.22	0.23	–	68.92	–	–	–	–	–
GS11-4	310.25	10.86	13.07	35.36	-	–	–	–	–	–	–	–
GS12-1	291.71	-	8.60	80.44	-	0.32	74.48	–	–	–	–	–
GS12-2	234.56	6.22	7.77	56.73	0.49	–	84.20	19.22%	15.50%	–	–	–
GS14-1	114.52	1.88	-	118.65	0.19	–	–	–	–	+	–	–
GS14-2	201.90	-	7.54	50.45	0.38	–	75.65	–	–	–	–	–
GS15-2	212.30	6.76	10.15	33.97	-	–	71.97	–	–	+	–	–

Each values is a mean of three independent trials.

^a^“I-P” means tricalcium phosphate, “O-P” means egg yolk lecithin.

^b^“t-Z” means trans-Zeatin, “GA_3_” means gibberellins, “IAA” means indoleacetic-3-acid, “−” means showed no detected on this hormones.

^c^1-aminocyclopropane-1-carboxylate deaminase activity.

^d^*“RS”* means *Rhizoctonia solani, “FO”* means *Fusarium oxysporum*.

^e^“−” means indicates the absence of PCR products and “+” means indicates the presence of PCR products for the phytase functional genes: *bpp*, β-propeller phytase; *hap*, histidine acid phytase; *cp*, cysteine phytase.

**FIGURE 3:**
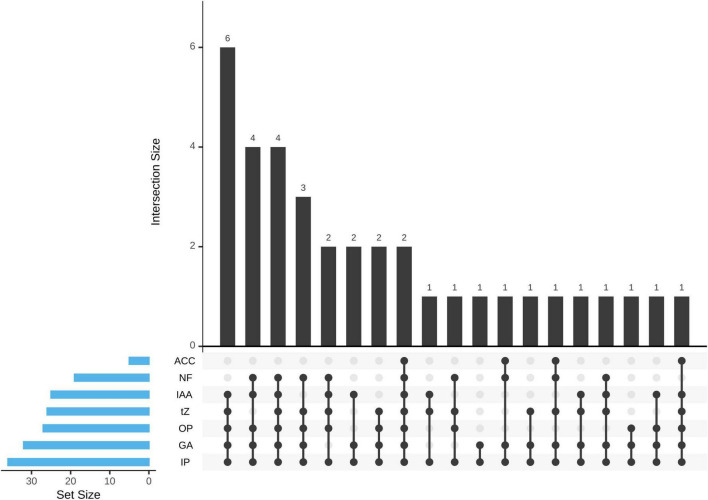
The upset plot representation of the 36 *Pseudomonas* sp. strains was allocated to seven different plant growth-promoting (PGP) traits. ACC, ACC deaminase activity; NF, nitrogen fixation; IAA, IAA production; tZ, t-Z production; OP, organic phosphate solubilization; GA, GA_3_ production; IP, inorganic phosphate solubilization.

The ability of PHs production (t-Z, GA_3_, IAA) was detected by HPLC. All the isolates can produce at least one or more PHs. Among them, a total of 15 isolates (41.67%) produce three, 17 isolates (47.22%) produce two, and 4 isolates (11.11%) produce one PH (Figure 3). A total of 26 isolates (72.22%) showed t-Z-producing ability in the range 3.98–17.45 μg mL^–1^, 32 isolates (88.89%) showed GA_3_-producing ability in the range 23.16–173.22 μg mL^–1^, and 25 isolates (69.44%) showed IAA-producing ability in the range 0.09–2.27 μg mL^–1^ (Table 1).

Only five isolates (13.89%) showed ACC deaminase activity in the range 0.32–3.14 μmol mg^–1^ protein h^–1^. The maximum activity of ACC deaminase by bacteria strains was observed in *Pseudomonas helmanticensis* GS8-2.

Nitrogen fixation of isolates was determined by indirect measurement of the amount of acetylene reduced to form ethylene. A total of 25 isolates (69.44%) were capable of growing on NFM medium and showed nitrogen fixation ability in the range of 51.53–94.01 nmol (C_2_H_4_) mg^–1^ protein h^–1^. *Pseudomonas helmanticensis* GS5-5 showed the highest nitrogenase activity, followed by *Pseudomonas graminis* GS3-2 (Table 1).

Antimicrobial activity of isolates of *R. solani* and *F. oxysporum* showed low inhibitory, as shown in Table 2. Eight isolates (22.22%) exhibited antimicrobial activity against *R. solani* in the range 6.89–25.94% and seven isolates (19.44%) against *F. oxysporum* in the range 8.60–32.90% (Table 1).

**TABLE 2:** Effects of strains inoculation on the growth of *Lolium perenne* L. seedlings.

Treatments	Shoot length (cm)	Root length (cm)	Shoot fresh weight (g plant^−1^)	Root fresh weight (g plant^−1^)	Root/Shoot ratio	Total root length (cm)	Root surface area (cm^2^)	Root volume (cm^3^)	Root average diameter (mm)
Control	14.01 ± 1.52d	21.52 ± 2.44a	0.071 ± 0.007e	0.168 ± 0.021c	2.37 ± 0.13a	265.15 ± 8.31a	20.61 ± 1.85b	0.127 ± 0.007c	0.247 ± 0.016e
GB5-5	17.69 ± 1.41c	8.80 ± 1.85e	0.079 ± 0.011d	0.136 ± 0.019e	1.99 ± 0.21d	53.28 ± 6.97g	6.22 ± 1.69f	0.058 ± 0.011f	0.371 ± 0.024ab
GS3-6	20.40 ± 1.01a	13.92 ± 1.51c	0.102 ± 0.009b	0.220 ± 0.028b	2.17 ± 0.15c	189.41 ± 8.48c	17.96 ± 2.22c	0.135 ± 0.039bc	0.302 ± 0.030c
GS6-5	18.83 ± 2.32b	13.42 ± 1.89c	0.091 ± 0.010c	0.137 ± 0.019e	1.70 ± 0.09e	162.87 ± 6.66d	14.63 ± 1.86d	0.105 ± 0.008d	0.286 ± 0.017d
GS10-1	20.65 ± 1.85a	17.61 ± 2.12b	0.137 ± 0.015a	0.307 ± 0.024a	2.24 ± 0.24bc	229.89 ± 14.98b	22.07 ± 2.09a	0.169 ± 0.027a	0.306 ± 0.049c
GS14-1	17.75 ± 0.69c	10.99 ± 1.31d	0.091 ± 0.013c	0.154 ± 0.026d	1.51 ± 0.10f	118.41 ± 5.54e	14.56 ± 2.28d	0.143 ± 0.018b	0.392 ± 0.023a
GS15-2	19.28 ± 1.75b	9.06 ± 0.78e	0.085 ± 0.008cd	0.157 ± 0.014d	1.84 ± 0.14d	87.17 ± 4.65f	9.19 ± 1.58e	0.077 ± 0.010e	0.336 ± 0.034b
LSD (*P* ≤ 0.05)	2.00	2.28	0.01	0.03	0.65	10.77	2.51	0.03	0.04

Each values is a mean ± SD of three independent trials.

The presence of functional genes related to phytase (*bpp*, *hap*, *cp*) was used to evaluate the enzyme class of isolates, the *bpp* gene was detected in six isolates by PCR amplification and confirmed by sequencing sequence alignment. The *hap* and *cp* gene PCR products showed non-specific amplification in this study (Table 1).

### Plant growth promotion potential under P-limitation

**FIGURE 4:**
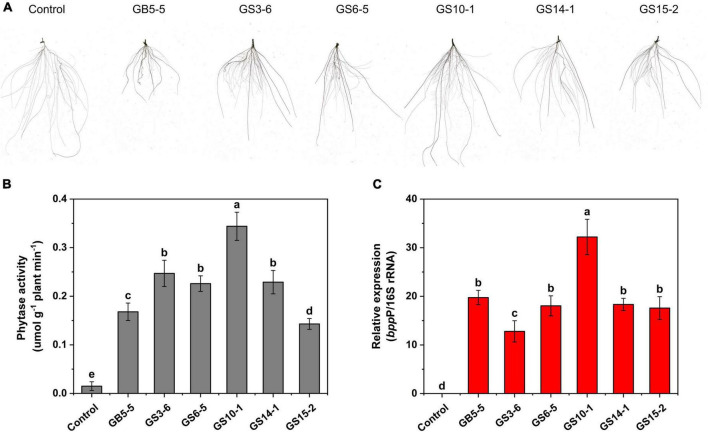
Effect of strains inoculation on *Lolium perenne* L. root system after 28 days for **(A)** root morphology, **(B)** root system phytase activity, and **(C)** phytase gene (*bpp*P) relative abundance. Control: root system of uninoculated strains. Each value is a mean ± SD of three independent trials.

The isolates (GB5-5, GS3-6, GS6-5, GS10-1, GS14-1, and GS15-2) were tested for their potential of growth-promoting for *Lolium perenne* L. when sodium phytate was the P source, and a non-inoculated control experiment was set. The strains had variable effects on the shoot and root in *Lolium perenne* L. All strains inoculation significantly enhanced the length and fresh weight of the shoot and reduced the root length to the control (*P* < 0.05). Among them, GS10-1 and GS3-6 increased by 82.74 and 30.95% the root fresh weight to the control, and also root/shoot ratio was the highest except for the control (*P* < 0.05). *Lolium perenne* L. promotes P uptake by regulating root morphology and configuration under P-limitation. In this study, all strains inoculation significantly (*P* < 0.05) reduced the total root length to the control (Figure 4A). Except for GS10-1, which was increased by 7.08%, the others significantly (*P* < 0.05) reduced the root surface area to the control. GS10-1 and GS14-1 were increased by 33.07 and 12.60% with the root volume. In addition, all treatments increased the root average diameter to the control. The highest growth promotion was GS10-1 compared to the others tested, which had significantly increased (*P* < 0.05) aboveground and underground biomass of *Lolium perenne* L. (Table 2).

We investigated the effect of strains inoculation on phytase activity in the *Lolium perenne* L. root system; the inoculation treatment significantly (*P* < 0.05) increased the root system activity to the control, among which GS10-1 showed the highest activity with 0.344 μmol g^–1^ plant min^–1^ (Figure 4B). In addition, we designed primers *bpp*P based on the conserved sequence of the phytase gene. The qPCR results showed that the phytase gene can be effectively expressed in the *Lolium perenne* L. root system (Figure 4C), and related to the results of root system phytase activity (*R*^2^ = 0.748; *P* < 0.05), GS10-1 showed highest relative expression of the phytase gene.

### Cloning and sequence analysis of phytase

**FIGURE 5:**
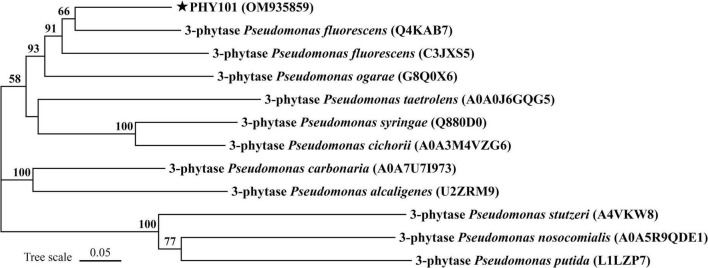
Neighbor-joining phylogenetic tree of PHY101 based on sequence similarity. The numbers at the nodes indicate the levels of bootstrap support based on data for 1,000 replicates. The scale bar represents 5% sequence divergence.

**FIGURE 6:**
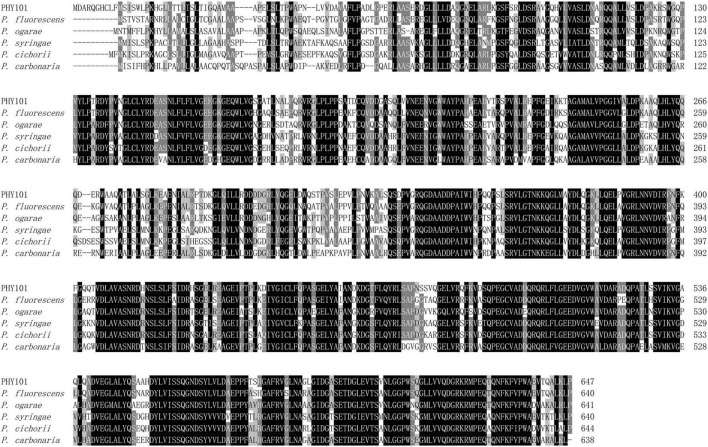
Multiple protein sequence alignment of phytase between PHY101 (OM935859) from *Pseudomonas mandelii* GS10-1 and other similarly species. The DNAMAN was utilized to perform the alignment for *Pseudomonas fluorescens* (Q4KAB7), *Pseudomonas fluorescens* (C3JXS5), *Pseudomonas ogarae* (G8Q0X6), *Pseudomonas syringae* (Q880D0), *Pseudomonas cichorii* (A0A3M4VZG6), and *Pseudomonas carbonaria* (A0A7U7I973). The consensus residues are identical in all sequences and the majority of sequences are shaded separately in a black and gray background.

The phytase PHY101 was amplified from the genome of *Pseudomonas mandelii* GS10-1 using gene-specific degenerate primers by PCR method. The ORF length of PHY101 was 1,941 bp and it encoded a polypeptide of 647 amino acids. The secreted proteins were predicted using TMHMM 2.0.^5^ The residue protein was a theoretical molecular mass of 70.1 kDa and a theoretical pI of 4.86. A UniProt BLAST search was performed using the deduced amino acid sequence and the identified gene/protein was a member of the phytase superfamily. The analysis of homologous amino acids revealed that PHY101 shared 79.4% similarity with 3-phytase (Q4KAB7) from *Pseudomonas fluorescens* and 75.9% similarity with 3-phytase (C3JXS5) from *Pseudomonas fluorescens* (Figure 5). The PHY101 was determined consisting of three parts based on the prediction of signal peptide and multiple alignments: a signal peptide of 35 amino acids, an inactive site N-terminal domain of 162 amino acids (66–228), and a BPPhy C-terminal domain of 325 amino acids. The alignment results of PHY101 with the five highest known homologous of closest sequences in UniProt are shown in Figure 6, in which the primary structure showed significant identities with *Pseudomonas* phytases and predicted it to be a typical BPPhy with six-bladed propeller shape by the 3D modeling results (Supplementary Figure 1).

### Production and purification of recombinant PHY101

**FIGURE 7:**
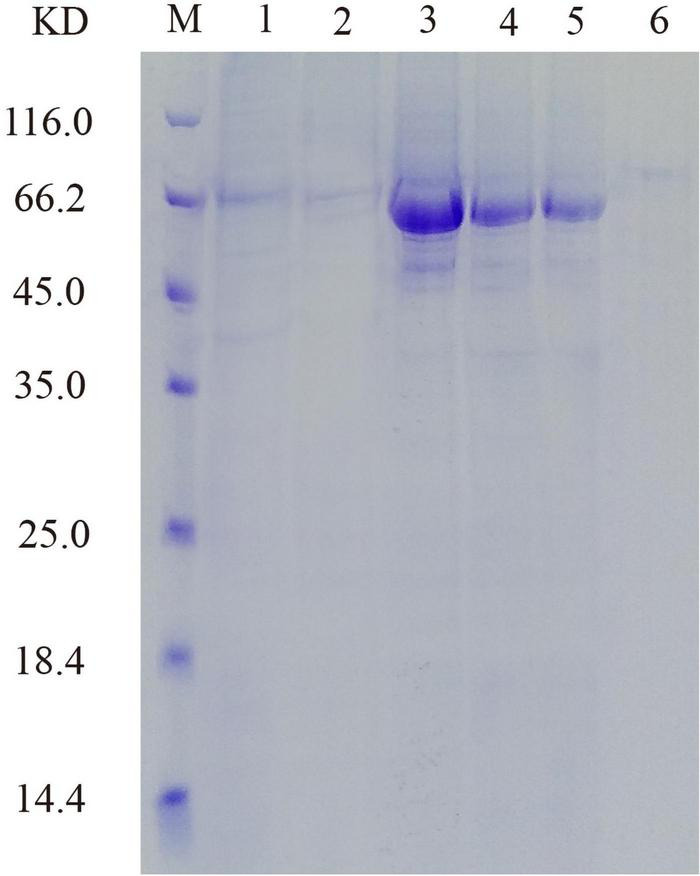
Sodium dodecyl sulfate-polyacrylamide gel electrophoresis (SDS-PAGE) analysis of the recombinant PHY101. M: Protein molecular weight marker; Lane 1: 96 h of culture to secrete supernatant; Lane 2: Effluent solution through column; Lane 3–6: Elution solution through column.

His-tagged versions of recombinant protein were successfully expressed and purified from the supernatant of cell lysate after induction, and SDS-PAGE showed that the molecular mass of PHY101 was around 67 kDa (without signal peptide) (Figure 7). The purified protein of PHY101 displayed a specific activity of 208 U mg^–1^ on sodium phytate.

### Biochemical profiles of recombinant PHY101

**FIGURE 8:**
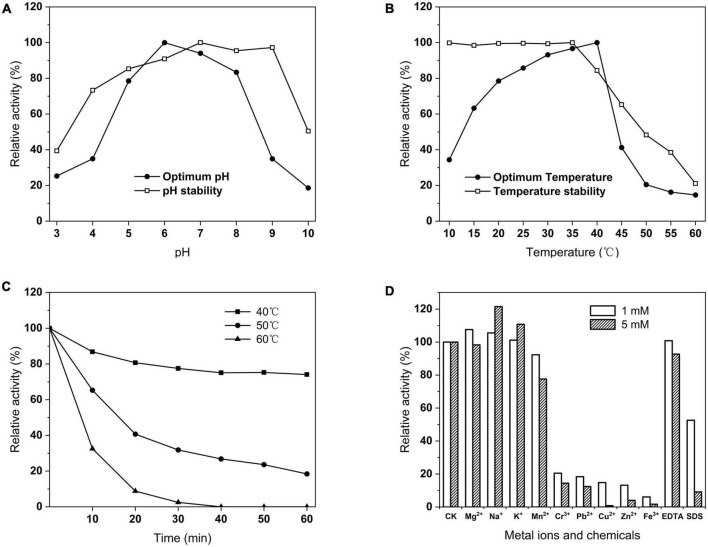
**(A)** Optimum and stability of pH on PHY101 activity from 3.0 to 10.0. **(B)** Optimum and stability of temperature on PHY101 activity from 10 to 60°C. **(C)** Thermal stability on PHY101 activity by different temperatures from 40 to 60°C for 10–60 min. **(D)** Effect of metal ions and chemical reagents on PHY101 activity. Each value is a mean of three independent trials.

Phytase activity was assayed at different pH and temperature conditions. The enzyme had more than 70% activity at pH 5–8 and was highest at pH 6 (Figure 8A). The stability of the enzyme at pH 4–9 was more than 60% after 1 h at 37°C (Figure 8A). The optimal temperature of the enzyme was 40°C, whereas a rapid decline with the temperature increases (Figure 8B). Notably, the enzyme still had 34.41% activity of its maximal activity at 10°C and 63.31% at 15°C. The activity of the enzyme was stably stable above 90% between 10 and 50°C at optimal pH (Figure 8B). The thermal stability results showed that the remaining activity of the enzyme was 69.09% at 40°C for 60 min, and 18.44% at 50°C for 60 min, and it lost all activity at 60°C for 40 min (Figure 8C). At optimal temperature and pH, Na^+^ and K^+^ ions have a stimulating effect on the PHY101 activity, 1 mM Mg^2+^ stimulated and 5 mM Mg^2+^ inhibited the activity. Respectively, Mn^2+^, EDTA slightly and Cr^3+^, Pb^2+^, Cu^2+^, Zn^2+^, Fe^3+^, SDS strongly inhibited of enzyme activity (Figure 8D).

## Discussion

In the next few decades, the required quantum of global grass production and P uptake will increase substantially (Sattari et al., 2016). Assuming no area expansion of grassland, the raising increase in grass yields can only be possible if the P status of the soil under grasslands is improved. On the other hand, although plants can only uptake inorganic orthophosphate anions, a considerable portion of P of grassland soil is present in the organic phosphorus (P_o_) form (Gerke, 2015; Menezes-Blackburn et al., 2018). Mineralization of the P_o_ forms requires these into plant available P through the action of phosphatase enzymes (Menezes-Blackburn et al., 2013). The PGPBs express extracellular phytases that hydrolyze recalcitrant phytate, which are excellent biotechnological substances and can be used as potential substitutes for P-fertilization (Zhuang et al., 2021). Therefore, understanding the PGPB isolates associated with the soil P cycle and conferring sustainability in agriculture to delineate their function in grasslands has become important.

All plant species may establish a relationship with some strains, and the selection of bacterial isolates able to mineralize phytate *in vitro* is an important and necessary step. In the present study, we used a combination of molecular biological and extracellular phytase activities for the selection of environmentally adaptable PGPBs from the rhizosphere soil of seven dominant types of grasses in the Qinghai-Tibet Plateau. The 16S rDNA sequencing analysis suggested 73 phytase-producing bacteria isolates grouped into two phyla which consisted of 16 different genera. Proteobacteria (90.41%) was the most dominant, including *Pseudomonas*, *Acinetobacter*, *Variovorax*, *Achromobacter*, *Pantoea*, *Arthrobacter*, etc., and more than 49% (36) of isolates belongs to *Pseudomonas*, which also confirmed that the γ-Proteobacteria was the main bacterial group producing phytase, as reported by Park et al. (2022). In addition, we compared the extracellular phytase activity between genera of *Pseudomonas* and others to confirm the dominant genus, and the results indicated that isolates of *Pseudomonas* in the present work had a higher average activity, which could be representative within strains colonized in grass rhizosphere in the soils studied. *Pseudomonas* was one of the common ecological groups in the rhizosphere of plants with various PGP traits such as dissolved soil insoluble P, fixed nitrogen, and secreted plant growth hormone (Zhuang et al., 2021). Previous literature has reported that some phosphorus solubilizing bacteria (PSB) belong to these genera (Bargaz et al., 2021; Li J. T. et al., 2021; Bi et al., 2022; Li et al., 2022), but most studies have not reported phytase production abilities. Our work suggests that screening by using calcium phytate as the P source provides an efficient approach to obtaining phytase-producing bacteria. Interestingly, no *Bacillus* species were identified in this work. *Bacillus* is the main group of soil microorganisms that secrete phytase, especially β-propeller phytases (BPPhy), and has been obtained and reported in different species (Jorquera et al., 2011; Zhao et al., 2021). Speculated reasons were due to the nutrients of the medium, the geographical environment differences, and the composition of plant species.

This study focuses on phytase, but it is evident that select PGPBs should be considered PGP traits that can be used in bio-fertilizers (Tchakounte et al., 2018), such as P-solubilization, plant hormones production, ACC deaminase activity, nitrogen fixation, and antimicrobial activity. In the present study, the genus of *Pseudomonas* isolates (36) was performed to text *in vitro* PGP characteristics, and we found that some isolates were multifunctional and showed no less than two or more traits, which were similar to the results of previous reports on PGPB research (Bargaz et al., 2021; Hazarika et al., 2021). Tricalcium phosphate and lecithin were commonly used as a medium for P sources to detect the ability of PGPB to dissolve organic/inorganic P (Wan et al., 2020). The amount of P solubilization by strains in this work was comparable to other PGPBs reported in the literature (Yahya et al., 2021). Moreover, all the strains showed an excellent solubilization ability on tricalcium phosphate, and a majority showed a digesting effect on lecithin, which was similar to that reported by Wan et al. (2020), the PSB harboring abilities in utilizing multiple P sources. Plant hormones (PHs) are a kind of trace organic compounds that play important roles in regulating plant growth, metabolism, and more. PHs produced by microorganisms mainly include indoleacetic-3-acid (IAA), gibberellic acid (GA_3_), and cytokinins (CTKs) (Streletskii et al., 2016). However, the previous method of PHs was mainly aimed at measuring IAA production, which has certain deficiencies in the detection accuracy and detection type. In the present study, we used HPLC to detect the contents of IAA, GA_3_, and t-Z in the strains’ fermentation products. The results showed that all strains to be tested can produce one or more PHs, but many strains produced far lower concentrations of IAA than the same species of other reports (Hazarika et al., 2021; Li M. Y. et al., 2021; Alotaibi et al., 2022), speculated that the reason is related to the different detection methods. HPLC is a world-recognized detection method for the detection of PHs and has the advantages of rapidity, sensitivity, high detection accuracy, and simultaneous determination of multiple components. However, studies have rarely reported on the characteristics of secreting different PHs using the HPLC method in PGPBs to our knowledge. Our method improves detection accuracy and expands the scope of detection range with PHs. ACC deaminase activity bacteria effectively protect plants against growth inhibition and make plants more resistant to pathogens, it is one of the indicators to determine the ability of PGP. We observed that few strains obtained in the present study contain ACC deaminase activity, and speculate that they are potentially related to less biotic or abiotic stresses in the environment of strains. For example, Li M. Y. et al. (2021) screened PGPB from the rhizosphere of four grass species in alpine grasslands of the Qilian Mountains, and their PGP properties results showed that 5 (7.46%) of the 67 isolates contain ACC deaminase. PGPB potentially converts free nitrogen in the air into compound nitrogen (ammonia/nitrate nitrogen) by nitrogen fixation which serves as a source of stimulating plant growth (Li et al., 2020; Alotaibi et al., 2022). They are divided into three kinds for abiogenous azotobacter, symbiotic nitrogen-fixing bacteria, and associative nitrogen-fixing bacteria, and *Pseudomonas* is a species of associative nitrogen-fixing bacteria (Mahmud et al., 2020). In the present study, most of the strains have the ability to fix nitrogen, with a little difference in nitrogenase activity, which is similar with the Hazarika et al. (2021), who reported that PGPB can escalate plant biomass by the accumulation of nitrogen. Soil microorganisms contained a few bacteria with antimicrobial activity. Eight antagonistic bacteria (22.22%) against *R. solani* and *F. oxysporum* were screened from all strains, which suggests that the *Pseudomonas* species have poor antibacterial activity in this study. These potentially related to the strains species, as reported by Penha et al. (2020) and Salazar et al. (2022) that *Bacillus* was the dominant antagonistic bacteria, and showed higher antibacterial activity than other species.

To enhance the understanding of phytase-encoding genes of PGPBs, we used degenerate PCR to amplify the phytase gene (*bpp*, *hap*, *cp*). Six strains (16.7%) were found positive for the *bpp* gene in PCR products. Previous literature showed that BPPs were the most widely distributed phytase in nature, and also the only phytase observed under neutral and alkaline pH conditions, in which optimal reaction conditions are similar to the rhizosphere environment (Jorquera et al., 2013; Cotta et al., 2016; Stout et al., 2016). Therefore, it is considered to be the most important phytase in the rhizosphere (Kumar et al., 2017). Our results also proved the above-reported conclusion. However, no *hap* and *cp* gene was amplified, the reason presumably is related to other pathways for the degradation of phytate or the existence of new undiscovered phytase encoding genes, and needs to be further studied.

To evaluate the growth-promoting performance of phytase-producing PGPB, and explain the process of P-metabolism of strains, we select *Lolium perenne* L. to measure the effect of strains inoculation on the growth of plants under P-limitation when sodium phytate was the P source, which is suitable for planting in cold regions (Ren et al., 2016). Strains inoculation produced beneficial functions to promote *Lolium perenne* L. growth, which significantly increased the aboveground biomass accumulation and the root system phytase activity. Similar to that reported by Barra et al. (2019), due to the Hoagland’s does not provide any plant available P in this work, the growth promoting of the plants may derived from the release of P by strains through mineralized phytates. Plant roots have response mechanisms such as root configuration changes (root hair length increase, density increase, root/shoot ratio increase, etc.) to improve the uptake efficiency of phosphate when they are under a P-deficient environment (Kavanova et al., 2006). In the present study, plants responded differently to strain inoculation, which is presumably related to the plant characteristics and the colonization ability of strains. Among them, *Pseudomonas mandelii* GS10-1 performed best and significantly increased the fresh weight, root surface area, root volume, and root average diameter of *Lolium perenne* L. In addition, qPCR proved that the *bpp* gene was effectively expressed in plant rhizosphere, and positively correlated with the root system phytase activity (*R*^2^ = 0.748). The primers designed by this study can be used for qPCR research of BPPs in *Pseudomonas* species. The qPCR verification of BPPs has been reported in several bacterial strains, such as Shen et al. (2016), who reported a BPP gene (phyPf) cloned from *Pseudomonas fluorescens* JZ-DZ1, where the results of qPCR proved that the secretion of phytase was regulated by phyPf. In particular, some reports indicate that the activity of microbial phosphatases was determined by the interaction between functional gene abundance, soil available P content, and substrate availability (Suleman et al., 2018; Rasul et al., 2021; Yahya et al., 2022). Therefore, screening of PGPBs with phytase genes can help identify the new microbial taxa capable of mineralizing organic P.

In order to reveal the basic characteristics of enzymes and discover novel phytases with desirable properties, the enzyme must be purified and identified. In recent years, a considerable number of phytases have been isolated and cloned in microorganisms, which have achieved heterologous expression, and their enzymatic properties are tested (Nam et al., 2014; Zhang et al., 2015; Rix et al., 2021). However, the phytases that possess high activity at low temperatures are still uncommon (Jang et al., 2018), which play a role in P cycling and potentially be suitable for use in cold areas. In the present study, we emphatically studied *Pseudomonas mandelii* GS10-1 and cloned a novel BPPhy (PHY101) with a high activity of phytase. The molecular weight of the recombinant protein was around 67 kDa, which is larger than most reported of BPPs. The optimal pH of PHY101 was 6.0, stable at pH 5.0–9.0, and the optimum temperature was 40°C, where the temperature is lower than most reported strains of *Pseudomonas* sp. (50°C) (Nam et al., 2014) and *Bacillus* sp. (55–60°C) (Borgi et al., 2014). In particular, the high activity of PHY101 at low temperatures (more than 60% of maximum enzyme activity at 15°C) is potentially suitable for being applied to agricultural production in the region since the temperature of the soil in alpine grasslands in below 20°C all year round (Kun, 2021). Strains growing in alpine habitat is a source of low temperature-tolerant enzymes. In addition, Jang et al. (2018) first reported a BPP with an N-terminal domain from *Pseudomonas* sp. FB15, and verified that the presence of the N-terminal domain improved the catalytic efficiency and low-temperature tolerance of BPPs (Jang et al., 2019). Kumar et al. (2017) reviewed the current developments in genes sources, diversity, biochemical properties, and structural elucidation of BPPhy, and reported and found that the C-terminal of BPPs is relatively conserved while the N-terminal motifs are more diverse, which is consistent with the results of our study findings. The effect of metal ions and chemical reagents on enzyme activity showed that Cr^3+^, Pb^2+^, Cu^2+^, Zn^2+^, and Fe^3+^ are potent inhibitors of PHY101 activity. According to Maenz and Classen (1998) reports, this inhibitory effect may be related to the formation of insoluble mineral-phytate complexes by phytate at neutral pH.

## Conclusion

This is the first report about combined phytase activity and plant growth-promoting traits for the screening of efficient PGPB candidates associated with alpine grasslands on the Qinghai-Tibetan Plateau. The phytase-producing PGPBs inoculated exhibited plant-growth promoting effect under P-limitation. Our results reveal the potential of selected indigenous bacteria to serve as biological inoculants for the restoration of degraded grasslands and P-deficient soils to enhance grass growth and productivity. Additionally, this study cloning and the function of a novel low-temperature resistance phytase form *Pseudomonas mandelii* GS10-1, which should be defined as a functional gene that participated in promoting plant growth. We make the case established that the alpine grasslands in the Qinghai-Tibetan Plateau are one of the effective sources of low temperature-tolerant active enzymes. Furthermore, future studies will focus on the use of whole genome sequencing and multi-omics technologies to study the composition, characteristics, and catalytic mechanisms of the bioactive compounds produced by those PGPBs, and the development of engineered rhizobacteria for practical applications.

## Data availability statement

The datasets presented in this study can be found in online repositories. The names of the repository/repositories and accession number(s) can be found in the article/Supplementary material.

## Author contributions

QL and JL designed the experiments and contributed to the writing and revision of the manuscript. QL and XY conducted all the experiments. QL, XY, and CL analyzed the data and wrote the manuscript. ML revised the manuscript. All authors contributed to the article and approved the submitted version.

## Footnotes

1
https://www.ezbiocloud.net

2
https://www.ncbi.nlm.nih.gov/

3
https://www.uniprot.org/

4
https://swissmodel.expasy.org/

5
https://services.healthtech.dtu.dk/

## References

[B1] Alori E. T. Glick B. R. Babalola O. O. (2017). Microbial phosphorus solubilization and its potential for use in sustainable agriculture. *Front. Microbiol.* 8:971. 10.3389/fmicb.2017.00971 28626450 PMC5454063

[B2] Alotaibi F. St-Arnaud M. Hijri M. (2022). In-depth characterization of plant growth promotion potentials of selected alkanes-degrading plant growth-promoting bacterial isolates. *Front. Microbiol.* 13:863702. 10.3389/fmicb.2022.863702 35422791 PMC9002309

[B3] Bae H. D. Yanke L. J. Cheng K. J. Selinger L. B. (1999). A novel staining method for detecting phytase activity. *J. Microbiol. Methods* 39 17–22. 10.1016/s0167-7012(99)00096-2 10579503

[B4] Bargaz A. Elhaissoufi W. Khourchi S. Benmrid B. Borden K. A. Rchiad Z. (2021). Benefits of phosphate solubilizing bacteria on belowground crop performance for improved crop acquisition of phosphorus. *Microbiol. Res.* 252:126842. 10.1016/j.micres.2021.126842 34438221

[B5] Barra P. J. Pontigo S. Delgado M. Parra-Almuna L. Duran P. Valentine A. J. (2019). Phosphobacteria inoculation enhances the benefit of P-fertilization on *Lolium perenne* in soils contrasting in P-availability. *Soil Biol. Biochem.* 136:107516. 10.1016/j.soilbio.2019.06.012

[B6] Bi W. X. Weng B. S. Yan D. H. Wang H. Wang M. K. Yan S. Y. (2022). Responses of phosphate-solubilizing microorganisms mediated phosphorus cycling to drought-flood abrupt alternation in summer maize field soil. *Front. Microbiol.* 12:768921. 10.3389/fmicb.2021.768921 35111138 PMC8802831

[B7] Borgi M. A. Boudebbouze S. Aghajari N. Szukala F. Pons N. Maguin E. (2014). The attractive recombinant phytase from *Bacillus licheniformis*: Biochemical and molecular characterization. *Appl. Microbiol. Biotechnol.* 98 5937–5947. 10.1007/s00253-013-5421-9 24337251

[B8] Cotta S. Cavalcante Franco Dias A. Seldin L. Andreote F. Van Elsas J. (2016). The diversity and abundance of phytase genes (β−propeller phytases) in bacterial communities of the maize rhizosphere. *Lett. Appl. Microbiol.* 62 264–268. 10.1111/lam.12535 26661994

[B9] Devi R. Kaur T. Kour D. Yadav A. Yadav A. N. Suman A. (2022). Minerals solubilizing and mobilizing microbiomes: A sustainable approach for managing minerals’ deficiency in agricultural soil. *J. Appl. Microbiol.* 133 1245–1272. 10.1111/jam.15627 35588278

[B10] Dhillon J. Torres G. Driver E. Figueiredo B. Raun W. R. (2017). World phosphorus use efficiency in cereal crops. *Agron. J.* 109 1670–1677. 10.2134/agronj2016.08.0483

[B11] Ding Y. Yi Z. Fang Y. He S. Li Y. He K. (2021). Multi-omics reveal the efficient phosphate-solubilizing mechanism of bacteria on rocky soil. *Front. Microbiol.* 12:761972. 10.3389/fmicb.2021.761972 34956124 PMC8696128

[B12] Dong L. Guo Q. Wang P. Zhang X. Su Z. Zhao W. (2020). Qualitative and quantitative analyses of the colonization characteristics of *Bacillus subtilis* strain NCD-2 on cotton root. *Curr. Microbiol.* 77 1600–1609. 10.1007/s00284-020-01971-y 32270206

[B13] Frank J. A. Reich C. I. Sharma S. Weisbaum J. S. Wilson B. A. Olsen G. J. (2008). Critical evaluation of two primers commonly used for amplification of bacterial 16S rRNA genes. *Appl. Environ. Microb.* 74 2461–2470. 10.1128/aem.02272-07 18296538 PMC2293150

[B14] George T. S. Giles C. D. Menezes-Blackburn D. Condron L. M. Gama-Rodrigues A. C. Jaisi D. (2018). Organic phosphorus in the terrestrial environment: A perspective on the state of the art and future priorities. *Plant Soil* 427 209–211. 10.1007/s11104-017-3488-2

[B15] Gerke J. (2015). Phytate (inositol hexakisphosphate) in soil and phosphate acquisition from inositol phosphates by higher plants. a review. *Plants Basel* 4 253–266. 10.3390/plants4020253 27135327 PMC4844319

[B16] Goh K. M. Shahar S. Chan K. G. Chong C. S. Amran S. I. Sani M. H. (2019). Current status and potential applications of underexplored prokaryotes. *Microorganisms* 7:468. 10.3390/microorganisms7100468 31635256 PMC6843859

[B17] Granada C. E. Passaglia L. M. De Souza E. M. Sperotto R. A. (2018). Is phosphorus solubilization the forgotten child of plant growth-promoting rhizobacteria? *Front. Microbiol.* 9:2054. 10.3389/fmicb.2018.02054 30233533 PMC6129612

[B18] Hardy R. W. Holsten R. D. Jackson E. K. Burns R. C. (1968). The acetylene-ethylene assay for N_2_ fixation: Laboratory and field evaluation. *Plant Physiol.* 43 1185–1207. 10.1104/pp.43.8.1185 16656902 PMC1086994

[B19] Haskett T. L. Tkacz A. Poole P. S. (2021). Engineering rhizobacteria for sustainable agriculture. *ISME J.* 15 949–964. 10.1038/s41396-020-00835-4 33230265 PMC8114929

[B20] Hazarika S. N. Saikia K. Borah A. Thakur D. (2021). Prospecting endophytic bacteria endowed with plant growth promoting potential isolated from *Camellia sinensis*. *Front. Microbiol.* 12:738058. 10.3389/fmicb.2021.738058 34659169 PMC8515050

[B21] Hu J. Yang T. Friman V. P. Kowalchuk G. A. Hautier Y. Li M. (2021). Introduction of probiotic bacterial consortia promotes plant growth via impacts on the resident rhizosphere microbiome. *P. Roy. Soc.* 288:20211396. 10.1098/rspb.2021.1396 34641724 PMC8511750

[B22] Huang H. Luo H. Yang P. Meng K. Wang Y. Yuan T. (2006). A novel phytase with preferable characteristics from *Yersinia intermedia*. *Biochem. Bioph. Res. Commun.* 350 884–889. 10.1016/j.bbrc.2006.09.118 17034758

[B23] Huang H. Shi P. Wang Y. Luo H. Shao N. Wang G. (2009). Diversity of beta-propeller phytase genes in the intestinal contents of grass carp provides insight into the release of major phosphorus from phytate in nature. *Appl. Environ. Microb.* 75 1508–1516. 10.1128/aem.02188-08 19151187 PMC2655465

[B24] Huang H. Q. Zhang R. Fu D. W. Luo J. J. Li Z. Y. Luo H. Y. (2011). Diversity, abundance and characterization of ruminal cysteine phytases suggest their important role in phytate degradation. *Environ. Microbiol.* 13 747–757. 10.1111/j.1462-2920.2010.02379.x 21105982

[B25] Jang W. J. Lee J. M. Hasan M. T. Kong I. S. (2019). Fusion of the N-terminal domain of *Pseudomonas* sp. phytase with *Bacillus* sp. phytase and its effects on optimal temperature and catalytic efficiency. *Enzyme Microb. Tech.* 126 69–76. 10.1016/j.enzmictec.2019.04.002 31000166

[B26] Jang W. J. Lee J. M. Park H. D. Choi Y. B. Kong I. S. (2018). N-terminal domain of the beta-propeller phytase of *Pseudomonas* sp. FB15 plays a role for retention of low-temperature activity and catalytic efficiency. *Enzyme Microb. Tech.* 117 84–90. 10.1016/j.enzmictec.2018.06.008 30037556

[B27] Jarosch K. A. Kandeler E. Frossard E. Bunemann E. K. (2019). Is the enzymatic hydrolysis of soil organic phosphorus compounds limited by enzyme or substrate availability? *Soil Biol. Biochem.* 139:107628. 10.1016/j.soilbio.2019.107628

[B28] Jorquera M. A. Crowley D. E. Marschner P. Greiner R. Fernandez M. T. Romero D. (2011). Identification of beta-propeller phytase-encoding genes in culturable *Paenibacillus* and *Bacillus* spp. from the rhizosphere of pasture plants on volcanic soils. *FEMS Microbiol. Ecol.* 75 163–172. 10.1111/j.1574-6941.2010.00995.x 21073489

[B29] Jorquera M. A. Gabler S. Inostroza N. G. Acuña J. J. Campos M. A. Menezes-Blackburn D. (2018). Screening and characterization of phytases from bacteria isolated from Chilean hydrothermal environments. *Microb. Ecol.* 75 387–399. 10.1007/s00248-017-1057-0 28861598

[B30] Jorquera M. A. Saavedra N. Maruyama F. Richardson A. E. Crowley D. E. del Catrilaf C. R. (2013). Phytate addition to soil induces changes in the abundance and expression of *Bacillus* β-propeller phytase genes in the rhizosphere. *FEMS Microbiol. Ecol.* 83 352–360. 10.1111/j.1574-6941.2012.01480.x 22928980

[B31] Kavanova M. Lattanzi F. A. Grimoldi A. A. Schnyder H. (2006). Phosphorus deficiency decreases cell division and elongation in grass leaves. *Plant Physiol.* 141 766–775. 10.1104/pp.106.079699 16648218 PMC1475472

[B32] Kumar V. Yadav A. N. Verma P. Sangwan P. Saxena A. Kumar K. (2017). β-Propeller phytases: Diversity, catalytic attributes, current developments and potential biotechnological applications. *Int. J. Biol. Macromol.* 98 595–609. 10.1016/j.ijbiomac.2017.01.134 28174082

[B33] Kun Y. (2021). *Data from: The Multiscale Observation Network of Soil Temperature and Moisture on the Central Tibetan Plateau (2010-2021).* Plateau: National Tibetan Plateau Data Center, 10.11888/Terre.tpdc.271918

[B34] Lei X. G. Weaver J. D. Mullaney E. Ullah A. H. Azain M. J. (2013). Phytase, a new life for an “Old” enyzme. *Annu. Rev. Anim. Biosci.* 1 283–309. 10.1146/annurev-animal-031412-103717 25387021

[B35] Li H. Y. Qiu Y. Z. Yao T. Ma Y. C. Zhang H. R. Yang X. L. (2020). Effects of PGPR microbial inoculants on the growth and soil properties of *Avena sativa*, *Medicago sativa*, and *Cucumis sativus* seedlings. *Soil Till. Res.* 199:104577. 10.1016/j.still.2020.104577

[B36] Li J. T. Lu J. L. Wang H. Y. Fang Z. Wang X. J. Feng S. W. (2021). A comprehensive synthesis unveils the mysteries of phosphate-solubilizing microbes. *Biol. Rev.* 96 2771–2793. 10.1111/brv.12779 34288351 PMC9291587

[B37] Li M. Cozzolino V. Mazzei P. Drosos M. Monda H. Hu Z. (2018). Effects of microbial bioeffectors and P amendements on P forms in a maize cropped soil as evaluated by P-31-NMR spectroscopy. *Plant Soil* 427 87–104. 10.1007/s11104-017-3405-8

[B38] Li M. Y. Wang J. L. Yao T. Wang Z. L. Zhang H. R. Li C. N. (2021). Isolation and characterization of cold-adapted PGPB and their effect on plant growth promotion. *J. Microbiol. Biotechn.* 31 1218–1230. 10.4014/jmb.2105.05012 34261854 PMC9705895

[B39] Li Y. Yu X. T. Zheng J. R. Gong Z. L. Xu W. L. (2022). Diversity and phosphate solubilizing characteristics of cultivable organophosphorus-mineralizing bacteria in the sediments of Sancha lake. *Int. J. Environ. Res. Public Health* 19:2320. 10.3390/ijerph19042320 35206506 PMC8872205

[B40] Lin L. Ling J. Peng Q. Lin X. Zhou W. Zhang Y. (2021). The distribution characteristics of β-propeller phytase genes in rhizosphere sediment provide insight into species specialty from phytic mineralization in subtropical and tropical seagrass ecosystems. *Ecotoxicology* 30 1781–1788. 10.1007/s10646-021-02425-2 34115256

[B41] Ma A. Zhang X. Jiang K. Zhao C. Liu J. Wu M. (2020). Phylogenetic and physiological diversity of cultivable actinomycetes isolated from alpine habitats on the Qinghai-Tibetan Plateau. *Front. Microbiol.* 11:555351. 10.3389/fmicb.2020.555351 33117304 PMC7566193

[B42] Maenz D. D. Classen H. L. (1998). Phytase activity in the small intestinal brush border membrane of the chicken. *Poultry Sci.* 77 557–563. 10.1093/ps/77.4.557 9565239

[B43] Mahmud K. Makaju S. Ibrahim R. Missaoui A. (2020). Current progress in nitrogen fixing plants and microbiome research. *Plants-Basel* 9:97. 10.3390/plants9010097 31940996 PMC7020401

[B44] Menezes-Blackburn D. Giles C. Darch T. George T. S. Blackwell M. Stutter M. (2018). Opportunities for mobilizing recalcitrant phosphorus from agricultural soils: A review. *Plant Soil* 427 5–16. 10.1007/s11104-017-3362-2 30996482 PMC6438637

[B45] Menezes-Blackburn D. Jorquera M. A. Greiner R. Gianfreda L. de la Luz Mora M. (2013). Phytases and phytase-labile organic phosphorus in manures and soils. *Crit. Rev. Environ. Sci. Tech.* 43 916–954. 10.1080/10643389.2011.627019

[B46] Nam S. J. Kim Y. O. Ko T. K. Kang J. K. Chun K. H. Auh J. H. (2014). Molecular and biochemical characteristics of beta-propeller phytase from marine *Pseudomonas* sp. BS10-3 and its potential application for animal feed additives. *J. Microbiol. Biotechnol.* 24 1413–1420. 10.4014/jmb.1407.07063 25112322

[B47] Nautiyal C. S. (1999). An efficient microbiological growth medium for screening phosphate solubilizing microorganisms. *FEMS Microbiol. Lett.* 170 265–270. 10.1111/j.1574-6968.1999.tb13383.x 9919677

[B48] Neal A. L. Blackwell M. Akkari E. Guyomar C. Clark I. Hirsch P. R. (2018). Phylogenetic distribution, biogeography and the effects of land management upon bacterial non-specific Acid phosphatase gene diversity and abundance. *Plant Soil* 427 175–189. 10.1007/s11104-017-3301-2 30996484 PMC6438641

[B49] Pankievicz V. C. S. do Amaral F. P. Ane J. M. Stacey G. (2021). Diazotrophic bacteria and their mechanisms to interact and benefit cereals. *Mol. Plant Microbe Interact.* 34 491–498. 10.1094/mpmi-11-20-0316-fi 33543986

[B50] Park Y. Solhtalab M. Thongsomboon W. Aristilde L. (2022). Strategies of organic phosphorus recycling by soil bacteria: Acquisition, metabolism, and regulation. *Env. Microbiol. Rep.* 14 3–24. 10.1111/1758-2229.13040 35001516 PMC9306846

[B51] Penha R. O. Vandenberghe L. P. S. Faulds C. Soccol V. T. Soccol C. R. (2020). *Bacillus lipopeptides* as powerful pest control agents for a more sustainable and healthy agriculture: Recent studies and innovations. *Planta* 251:70. 10.1007/s00425-020-03357-7 32086615

[B52] Penrose D. M. Glick B. R. (2003). Methods for isolating and characterizing ACC deaminase-containing plant growth-promoting rhizobacteria. *Physiol. Plant.* 118 10–15. 10.1034/j.1399-3054.2003.00086.x 12702008

[B53] Perrig D. Boiero M. L. Masciarelli O. A. Penna C. Ruiz O. A. Cassan F. D. (2007). Plant-growth-promoting compounds produced by two agronomically important strains of *Azospirillum brasilense*, and implications for inoculant formulation. *Appl. Microbiol. Biotechnol.* 75 1143–1150. 10.1007/s00253-007-0909-9 17345081

[B54] Ramakrishna N. Lacey J. Smith J. E. (1991). Effect of surface sterilization, fumigation and gamma irradiation on the microflora and germination of barley seeds. *Int. J. Food Microbiol.* 13 47–54. 10.1016/0168-1605(91)90135-c 1863528

[B55] Rasul M. Yasmin S. Yahya M. Breitkreuz C. Tarkka M. Reitz T. (2021). The wheat growth-promoting traits of *Ochrobactrum* and *Pantoea* species, responsible for solubilization of different P sources, are ensured by genes encoding enzymes of multiple P-releasing pathways. *Microbiol. Res.* 246:126703. 10.1016/j.micres.2021.126703 33482437

[B56] Ren Y. X. Liu Y. Sun J. F. Lu H. Yang L. Chen C. C. (2016). *Lolium perenne* as the cultivation plant in hydroponic ditch and constructed wetland to improve wastewater treatment efficiency in a cold region. *Wetlands* 36 659–665. 10.1007/s13157-016-0776-1

[B57] Rix G. D. Todd J. D. Neal A. L. Brearley C. A. (2021). Improved sensitivity, accuracy and prediction provided by a high-performance liquid chromatography screen for the isolation of phytase-harbouring organisms from environmental samples. *Microb. Biotechnol.* 14 1409–1421. 10.1111/1751-7915.13733 33347708 PMC8313252

[B58] Saitou N. Nei M. (1987). The neighbor-joining method: A new method for reconstructing phylogenetic trees. *Mol. Biol. Evol.* 4 406–425. 10.1093/oxfordjournals.molbev.a040454 3447015

[B59] Salazar B. Ortiz A. Keswani C. Minkina T. Mandzhieva S. Singh S. P. (2022). *Bacillus* spp. as bio-factories for antifungal secondary metabolites: Innovation beyond whole organism formulations. *Microb. Ecol.* [Epub ahead of print]. 10.1007/s00248-022-02044-2 35604432

[B60] Sattari S. Z. Bouwman A. F. Rodriguez R. M. Beusen A. H. W. Van Ittersum M. K. (2016). Negative global phosphorus budgets challenge sustainable intensification of grasslands. *Nat. Commun.* 7:10696. 10.1038/ncomms10696 26882144 PMC4757762

[B61] Shen L. Wu X. Q. Zeng Q. W. Liu H. B. (2016). Regulation of soluble phosphate on the ability of phytate mineralization and β-Propeller phytase gene expression of *Pseudomonas fluorescens* JZ-DZ1, a phytate-mineralizing rhizobacterium. *Curr. Microbiol.* 73 915–923. 10.1007/s00284-016-1139-0 27664014

[B62] Singh B. Boukhris I. Kumar V. Yadav A. N. Farhat-Khemakhem A. Kumar A. (2020). Contribution of microbial phytases to the improvement of plant growth and nutrition: A review. *Pedosphere* 30 295–313. 10.1016/S1002-0160(20)60010-8

[B63] Spector T. (1978). Refinement of the coomassie blue method of protein quantitation. A simple and linear spectrophotometric assay for less than or equal to 0.5 to 50 microgram of protein. *Anal. Biochem.* 86 142–146. 10.1016/0003-2697(78)90327-5 655375

[B64] Stout L. M. Nguyen T. T. Jaisi D. P. (2016). Relationship of phytate, phytate-mineralizing bacteria, and beta-propeller phytase genes along a coastal tributary to the Chesapeake Bay. *Soil Sci. Soc. Am. J.* 80 84–96. 10.2136/sssaj2015.04.0146

[B65] Streletskii R. A. Kachalkin A. V. Glushakova A. M. Demin V. V. Chernov I. Y. (2016). Quantitative determination of indole-3-acetic acid in yeasts using high performance liquid chromatography-tandem mass spectrometry. *Microbiology* 85 727–736. 10.1134/s0026261716060187

[B66] Stromberg C. A. E. Staver A. C. (2022). The history and challenge of grassy biomes. *Science* 377 592–593. 10.1126/science.add1347 35926015

[B67] Suleman M. Yasmin S. Rasul M. Yahya M. Atta B. M. Mirza M. S. (2018). Phosphate solubilizing bacteria with glucose dehydrogenase gene for phosphorus uptake and beneficial effects on wheat. *PLoS One* 13:e0204408. 10.1371/journal.pone.0204408 30240432 PMC6150522

[B68] Tapia-García E. Y. Hernández-Trejo V. Guevara-Luna J. Rojas-Rojas F. U. Arroyo-Herrera I. Meza-Radilla G. (2020). Plant growth-promoting bacteria isolated from wild legume nodules and nodules of *Phaseolus vulgaris* L. trap plants in central and southern Mexico. *Microbiol. Res.* 239:126522. 10.1016/j.micres.2020.126522 32585580

[B69] Tchakounte G. V. T. Berger B. Patz S. Fankem H. Ruppel S. (2018). Community structure and plant growth-promoting potential of cultivable bacteria isolated from Cameroon soil. *Microbiol. Res.* 214 47–59. 10.1016/j.micres.2018.05.008 30031481

[B70] Turner B. L. Paphazy M. J. Haygarth P. M. McKelvie I. D. (2002). Inositol phosphates in the environment. *Philos. Trans. R. Soc. Lond. B. Biol. Sci.* 357 449–469. 10.1098/rstb.2001.0837 12028785 PMC1692967

[B71] Vasseur-Coronado M. du Boulois H. D. Pertot I. Puopolo G. (2021). Selection of plant growth promoting rhizobacteria sharing suitable features to be commercially developed as biostimulant products. *Microbiol. Res.* 245:126672. 10.1016/j.micres.2020.126672 33418398

[B72] Wan W. Qin Y. Wu H. Zuo W. He H. Tan J. (2020). Isolation and characterization of phosphorus solubilizing bacteria with multiple phosphorus sources utilizing capability and their potential for lead immobilization in soil. *Front. Microbiol.* 11:752. 10.3389/fmicb.2020.00752 32390988 PMC7190802

[B73] Yahya M. Islam E. U. Rasul M. Farooq I. Mahreen N. Tawab A. (2021). Differential root exudation and architecture for improved growth of wheat mediated by phosphate solubilizing bacteria. *Front. Microbiol.* 12:744094. 10.3389/fmicb.2021.744094 34721342 PMC8554232

[B74] Yahya M. Rasul M. Sarwar Y. Suleman M. Tariq M. Hussain S. Z. (2022). Designing synergistic biostimulants formulation containing autochthonous phosphate-solubilizing bacteria for sustainable wheat production. *Front. Microbiol.* 13:889073. 10.3389/fmicb.2022.889073 35592004 PMC9111743

[B75] Yao Q. Li Z. Song Y. Wright S. J. Guo X. Tringe S. G. (2018). Community proteogenomics reveals the systemic impact of phosphorus availability on microbial functions in tropical soil. *Nat. Ecol. Evol.* 2 499–509. 10.1038/s41559-017-0463-5 29358607

[B76] Yoon S. H. Ha S. M. Kwon S. Lim J. Kim Y. Seo H. (2017). Introducing EzBioCloud: A taxonomically united database of 16S rRNA gene sequences and whole-genome assemblies. *Int. J. Syst. Evol. Microbiol.* 67 1613–1617. 10.1099/ijsem.0.001755 28005526 PMC5563544

[B77] Zhang S. Liao S. A. Yu X. Lu H. Xian J. A. Guo H. (2015). Microbial diversity of mangrove sediment in Shenzhen Bay and gene cloning, characterization of an isolated phytase-producing strain of SPC09 *B. cereus*. *Appl. Microbiol. Biotechnol.* 99 5339–5350. 10.1007/s00253-015-6405-8 25646962

[B78] Zhao T. Yong X. Zhao Z. Dolce V. Li Y. Curcio R. (2021). Research status of *Bacillus* phytase. *3 Biotech* 11:415. 10.1007/s13205-021-02964-9 34485008 PMC8377137

[B79] Zhuang L. Li Y. Wang Z. Yu Y. Zhang N. Yang C. (2021). Synthetic community with six *Pseudomonas* strains screened from garlic rhizosphere microbiome promotes plant growth. *Microb. Biotechnol.* 14 488–502. 10.1111/1751-7915.13640 32762153 PMC7936309

