# Corrigendum: In-depth characterization of phytase-producing plant growth promotion bacteria isolated in alpine grassland of Qinghai-Tibetan Plateau

**DOI:** 10.3389/fmicb.2023.1173834

**Published:** 2023-03-13

**Authors:** Qi Li, Xiaolei Yang, Jianhong Li, Mingyuan Li, Changning Li, Tuo Yao

**Affiliations:** Key Laboratory of Grassland Ecosystem, Ministry of Education, Sino-U.S. Centers for Grazing Land Ecosystem Sustainability, Ministry of Science and Technology, College of Pratacultural Science, Gansu Agricultural University, Lanzhou, China

**Keywords:** phytase, plant growth-promoting bacteria, plant growth promotion, β-propeller phytase, *Pseudomonas mandelii* GS10-1, heterologous expression

In the published article, there was an error in the **Funding** statement. We found that Funding part in the published articles was wrong in “This study was financially supported by grants from the Education Science and Technology Innovation Project of Gansu Province (grant number GSSYLXM-02) and the Fertilizer Production and Demonstration Based on Livestock and Poultry Waste in Gansu Province (grant number 2020 C-16),” which was due to our negligence in submitting the revised papers. The correct **Funding** statement appears below.

