# Supplementary material for: In-depth characterization of phytase-producing plant growth promotion bacteria isolated in alpine grassland of Qinghai-Tibetan Plateau
Source: Front Microbiol. 2023 Jan 4;13:1019383. doi: 10.3389/fmicb.2022.1019383 (PMC9846362; doi:10.3389/fmicb.2022.1019383)

**Supplementary Table S1** Identification of phytase-producing bacteria based on 16S rRNA gene sequences similarity and its phytase activity.

| **Isolate no.** | **Media** | **Host plant** | **Most similar strain (ID)** | **%**  **Similarity** | **GenBank accession no.** | **Phytase activity**  **(U mL^-1^)** |
| --- | --- | --- | --- | --- | --- | --- |
| GB1-9 | M1 | *Bupleurum chinense* | *Pseudomonas brassicacearum* subsp. *neoaurantiaca* ATCC 49054 (EU391388) | 99.71 | OP412502 | 0.259±0.028 |
| GB2-1 | M1 | *Stipa capillata* | *Acinetobacter calcoaceticus* DSM 30006 (AIEC01000170) | 100.00 | OP412532 | 0.33±0.048 |
| GB2-2 | M1 | *Astragalus chilienshanensis* | *Pantoea eucalypti* LMG 24198 (EF688009) | 99.85 | OP412559 | 0.12±0.022 |
| GB2-3 | M1 | *Bupleurum chinense* | *Acinetobacter calcoaceticus* DSM 30006 (AIEC01000170) | 99.78 | OP412533 | 0.35±0.036 |
| GB2-8 | M1 | *Stipa capillata* | *Pseudomonas fluorescens* DSM 50090 (LHVP01000014) | 99.85 | OP412503 | 0.271±0.033 |
| GB2-9 | M1 | *Avena sativa* | *Massilia arenae* GEM5 (KT369857) | 99.56 | OP412560 | 0.108±0.018 |
| GB3-1 | M1 | *Poa pratensis* | *Pseudomonas atacamensis* M7D1 (SSBS01000008) | 99.93 | OP412507 | 0.191±0.02 |
| GB3-4 | M1 | *Kobresia kansuensis* | *Pseudomonas psychrophila* E-3 (AB041885) | 100.00 | OP412508 | 0.327±0.019 |
| GB4-1 | M1 | *Stipa capillata* | *Arthrobacter pascens* DSM 20545 (X80740) | 99.63 | OP412558 | 0.131±0.021 |
| GB4-6 | M1 | *Stipa capillata* | *Microbacterium oxydans* DSM 20578 (Y17227) | 99.78 | OP412568 | 0.137±0.028 |
| GB4-8 | M1 | *Bupleurum chinense* | *Microbacterium oxydans* DSM 20578 (Y17227) | 100.00 | OP412569 | 0.213±0.025 |
| GB5-2 | M1 | *Stipa capillata* | *Variovorax paradoxus* NBRC 15149 (BCUT01000013) | 99.71 | OP412527 | 0.097±0.03 |
| GB5-4 | M1 | *Bupleurum chinense* | *Microbacterium paraoxydans* NBRC 103076 (BCRH01000180) | 99.78 | OP412562 | 0.14±0.033 |
| GB5-5 | M1 | *Avena sativa* | *Pseudomonas canadensis* 2-92 (AYTD01000015) | 100.00 | OP412530 | 0.377±0.023 |
| GB5-6 | M1 | *Avena sativa* | *Achromobacter deleyi* LMG 3458 (HG324053) | 99.63 | OP412525 | 0.21±0.025 |
| GB5-8 | M1 | *Stipa capillata* | *Achromobacter marplatensis* B2 (EU150134) | 99.85 | OP412526 | 0.109±0.037 |
| GB6-2 | M1 | *Stipa capillata* | *Variovorax paradoxus* NBRC 15149 (BCUT01000013) | 100.00 | OP412528 | 0.174±0.017 |
| GB6-6 | M1 | *Poa pratensis* | *Pseudomonas simiae* OLi (AJ936933) | 100.00 | OP412531 | 0.225±0.018 |
| GB7-3 | M1 | *Poa pratensis* | *Acinetobacter calcoaceticus* DSM 30006 (AIEC01000170) | 100.00 | OP412534 | 0.107±0.017 |
| GB7-8 | M1 | *Astragalus chilienshanensis* | *Variovorax boronicumulans* BAM-48 (AB300597) | 99.71 | OP412529 | 0.127±0.035 |
| GB8-1 | M1 | *Avena sativa* | *Acinetobacter calcoaceticus* DSM 30006 (AIEC01000170) | 100.00 | OP412535 | 0.212±0.02 |
| GB8-5 | M1 | *Avena sativa* | *Acinetobacter kookii* ANC 4667 (jgi.1102384) | 98.90 | OP412536 | 0.243±0.023 |
| GB8-9 | M1 | *Bupleurum chinense* | *Rahnella aceris* SAP-19 (MN737201) | 100.00 | OP412537 | 0.183±0.025 |
| GB9-1 | M1 | *Bupleurum chinense* | *Pseudomonas mandelii* NBRC 103147 (BDAF01000092) | 99.78 | OP412542 | 0.243±0.019 |
| GB9-2 | M1 | *Bupleurum chinense* | *Pseudomonas vancouverensis* ATCC 700688 (AJ011507) | 99.78 | OP412541 | 0.329±0.021 |
| GB9-3 | M1 | *Bupleurum chinense* | *Pantoea agglomerans* DSM 3493 (AJ233423) | 99.41 | OP412540 | 0.163±0.011 |
| GB9-4 | M1 | *Oxytropis ochrocephala* | *Enterobacter hormaechei* subsp. *hoffmannii* EN-114 (CP017186) | 99.78 | OP412543 | 0.268±0.033 |
| GB10-2 | M1 | *Stipa capillata* | *Phyllobacterium ifriqiyense* STM 370 (AY785325) | 99.77 | OP412546 | 0.134±0.022 |
| GB10-3 | M1 | *Astragalus chilienshanensis* | *Phyllobacterium trifolii* PETP02 (AY786080) | 99.39 | OP412545 | 0.285±0.041 |
| GB10-6 | M1 | *Bupleurum chinense* | *Phyllobacterium ifriqiyense* STM 370 (AY785325) | 100.00 | OP412547 | 0.129±0.015 |
| GB11-1 | M1 | *Stipa capillata* | *Phyllobacterium ifriqiyense* STM 370 (AY785325) | 99.92 | OP412548 | 0.098±0.026 |
| GB12-9 | M1 | *Stipa capillata* | *Pseudomonas neuropathica* P155 (LR797591) | 99.76 | OP412505 | 0.144±0.041 |
| GB14-2 | M1 | *Kobresia kansuensis* | *Pseudomonas umsongensis* DSM 16611 (NIWU01000003) | 99.78 | OP412506 | 0.220±0.042 |
| GS1-3 | M2 | *Astragalus chilienshanensis* | *Variovorax boronicumulans* BAM-48 (AB300597) | 100.00 | OP412561 | 0.126±0.054 |
| GS1-6 | M2 | *Stipa capillata* | *Pseudomonas migulae* CIP 105470 (AF074383) | 99.93 | OP412523 | 0.341±0.019 |
| GS1-7 | M2 | *Bupleurum chinense* | *Paenarthrobacter aurescens* NBRC 12136 (BJMD01000050) | 99.70 | OP412551 | 0.115±0.049 |
| GS1-8 | M2 | *Astragalus chilienshanensis* | *Pseudomonas kitaguniensis* MAFF 301498 (LC500862) | 100.00 | OP412522 | 0.262±0.028 |
| GS2-1 | M2 | *Kobresia kansuensis* | *Pseudomonas helmanticensis* OHA11 (HG940537) | 99.71 | OP412521 | 0.217±0.019 |
| GS2-4 | M2 | *Stipa capillata* | *Rahnella victoriana* FRB 225 (KF308403) | 99.55 | OP412538 | 0.239±0.027 |
| GS2-5 | M2 | *Poa pratensis* | *Pseudomonas brassicacearum* subsp. *neoaurantiaca* ATCC 49054 (EU391388) | 99.85 | OP412555 | 0.138±0.02 |
| GS2-6 | M2 | *Oxytropis ochrocephala* | *Ensifer meliloti* LMG 6133 (X67222) | 100.00 | OP412550 | 0.11±0.021 |
| GS2-7 | M2 | *Avena sativa* | *Microbacterium oxydans* DSM 20578 (Y17227) | 100.00 | OP412563 | 0.178±0.013 |
| GS2-9 | M2 | *Avena sativa* | *Brucella pituitosa* CCUG 50899 (AM490609) | 99.85 | OP412549 | 0.088±0.022 |
| GS3-1 | M2 | *Kobresia kansuensis* | *Acinetobacter pseudolwoffii* ANC 5044 (PHRG01000001) | 98.90 | OP412557 | 0.333±0.033 |
| GS3-2 | M2 | *Stipa capillata* | *Pseudomonas graminis* DSM 11363 (Y11150) | 99.78 | OP412554 | 0.236±0.017 |
| GS3-6 | M2 | *Kobresia kansuensis* | *Pseudomonas mandelii* NBRC 103147 (BDAF01000092) | 99.71 | OP412497 | 0.146±0.015 |
| GS3-7 | M2 | *Bupleurum chinense* | *Pseudomonas izuensis* IzPS43-3003 (MN865785) | 99.78 | OP412553 | 0.37±0.04 |
| GS4-1 | M2 | *Bupleurum chinense* | *Pseudomonas neuropathica* P155 (LR797591) | 99.84 | OP412520 | 0.297±0.021 |
| GS4-2 | M2 | *Kobresia kansuensis* | *Pseudomonas izuensis* IzPS43_3003 (MN865785) | 99.78 | OP412509 | 0.23±0.022 |
| GS5-1 | M2 | *Kobresia kansuensis* | *Pantoea vagans* LMG 24199 (EF688012) | 99.70 | OP412539 | 0.343±0.025 |
| GS5-2 | M2 | *Astragalus chilienshanensis* | *Pararhizobium herbae* CCBAU 83011 (GU565534) | 99.70 | OP412544 | 0.055±0.009 |
| GS5-5 | M2 | *Astragalus chilienshanensis* | *Pseudomonas helmanticensis* OHA11 (HG940537) | 99.93 | OP412519 | 0.241±0.041 |
| GS6-2 | M2 | *Stipa capillata* | *Pseudomonas mandelii* NBRC 103147 (BDAF01000092) | 99.71 | OP412498 | 0.311±0.025 |
| GS6-3 | M2 | *Oxytropis ochrocephala* | *Ensifer morelensis* Lc04 (AY024335) | 99.62 | OP412566 | 0.169±0.034 |
| GS6-5 | M2 | *Avena sativa* | *Pseudomonas baetica* a390 (FM201274) | 100.00 | OP412510 | 0.391±0.018 |
| GS6-9 | M2 | *Kobresia kansuensis* | *Raoultella planticola* ATCC 33531 (JMPP01000074) | 99.85 | OP412567 | 0.152±0.009 |
| GS7-2 | M2 | *Bupleurum chinense* | *Pseudomonas mandelii* NBRC 103147 (BDAF01000092) | 99.78 | OP412499 | 0.292±0.043 |
| GS7-3 | M2 | *Oxytropis ochrocephala* | *Pseudomonas baetica* a390 (FM201274) | 99.93 | OP412518 | 0.388±0.018 |
| GS8-2 | M2 | *Bupleurum chinense* | *Pseudomonas helmanticensis* OHA11 (HG940537) | 99.71 | OP412517 | 0.255±0.014 |
| GS9-2 | M2 | *Astragalus chilienshanensis* | *Pseudomonas neuropathica* P155 (LR797591) | 99.76 | OP412516 | 0.256±0.02 |
| GS9-3 | M2 | *Astragalus chilienshanensis* | *Pseudomonas neuropathica* P155 (LR797591) | 99.84 | OP412512 | 0.077±0.027 |
| GS10-1 | M2 | *Avena sativa* | *Pseudomonas mandelii* NBRC 103147 (BDAF01000092) | 99.71 | OP412552 | 0.387±0.018 |
| GS11-2 | M2 | *Bupleurum chinense* | *Pseudomonas helmanticensis* OHA11 (HG940537) | 99.71 | OP412515 | 0.25±0.013 |
| GS11-3 | M2 | *Kobresia kansuensis* | *Pseudomonas edaphica* RD25 (LC272923) | 99.85 | OP412504 | 0.298±0.027 |
| GS11-4 | M2 | *Stipa capillata* | *Pseudomonas alloputida* Kh7 (LT718459) | 99.63 | OP412524 | 0.243±0.013 |
| GS12-1 | M2 | *Kobresia kansuensis* | *Pseudomonas mandelii* NBRC 103147 (BDAF01000092) | 99.63 | OP412500 | 0.124±0.044 |
| GS12-2 | M2 | *Avena sativa* | *Pseudomonas neuropathica* P155 (LR797591) | 99.76 | OP412514 | 0.155±0.037 |
| GS12-6 | M2 | *Poa pratensis* | *Arthrobacter rhombi* F.98.3HR.69 (Y15885) | 99.56 | OP412564 | 0.16±0.022 |
| GS12-9 | M2 | *Kobresia kansuensis* | *Achromobacter marplatensis* B2 (EU150134) | 99.85 | OP412556 | 0.071±0.03 |
| GS13-1 | M2 | *Poa pratensis* | *Pseudomonas reinekei* Mt-1 (AM293565) | 99.78 | OP412513 | 0.366±0.015 |
| GS14-2 | M2 | *Oxytropis ochrocephala* | *Pseudomonas donghuensis* HYS (AJJP01000212) | 100.00 | OP412511 | 0.268±0.021 |
| GS15-2 | M2 | *Oxytropis ochrocephala* | *Pseudomonas mandelii* NBRC 103147 (BDAF01000092) | 99.63 | OP412501 | 0.271±0.033 |
| GS16-2 | M2 | *Stipa capillata* | *Raoultella terrigena* ATCC 33257 (Y17658) | 99.49 | OP412565 | 0.21±0.021 |

**Supplementary Table S2** PCR primers sets used in this study.

| Gene target | Primer | Sequence (5′-3′) | Amplicon  size (bp) | Reference |
| --- | --- | --- | --- | --- |
| β-propeller phytase (BPPhy) | *bpp*-F | GAC GCA GCC GA Y GA Y CCN GCN ITN TGG | 160-200 | (Huang et al., 2009) |
|  | *bpp*-R | CAG GSC GCA NRT CIA CRT TRT T |  |  |
| histidine acid phytase (HAPhy) | *hap*-F | GTK STK AWW KTG AGY CGC CA | 800-900 | (Huang et al., 2011) |
|  | *hap*-R | TWK GCM AKR TTR GTA TCA TG |  |  |
| cysteine phytase (CPhy) | *cp*-F | GTG GAC CTR CGR MAR GAR WCI CA | 380-340 | (Huang et al., 2006) |
|  | *cp*-R | GTC CGA CCA TTG CCT GCY TCR ART GRA MRT GIA DCC |  |  |
| qPCR (*Pseudomonas* sp. BPPhy) | *bpp*P-F | TTG GAT TCA CCC GCA GCA A | ~80 | This study |
|  | *bpp*P-R | CAG GTC GTA CGC CAT CAG TC |  |  |
| BPPhy left-wing sequence PCR | LB-SP1 | GGA TTC ACC CGC AGC AAC CGT | / | This study |
|  | LB-SP2 | AAG CAG GGA CTG ATG GCG TAC GAC C |  |  |
|  | LB-SP3 | CCG GTG GGG CGC CTG AAC AAT GTC |  |  |
| BPPhy right-wing sequence PCR | RB-SP1 | GCG CAA GTC GAC ATT GTT CAG | / | This study |
|  | RB-SP2 | TGC CTT GCA GGT CGT ACG CCA TCA G |  |  |
|  | RB-SP3 | GTG CCC AGT ACA CGG CTC AGC GAC |  |  |

**Supplementary Figure S1** 3D model of the PHY101 catalytic domain built with SWISS-MODEL.


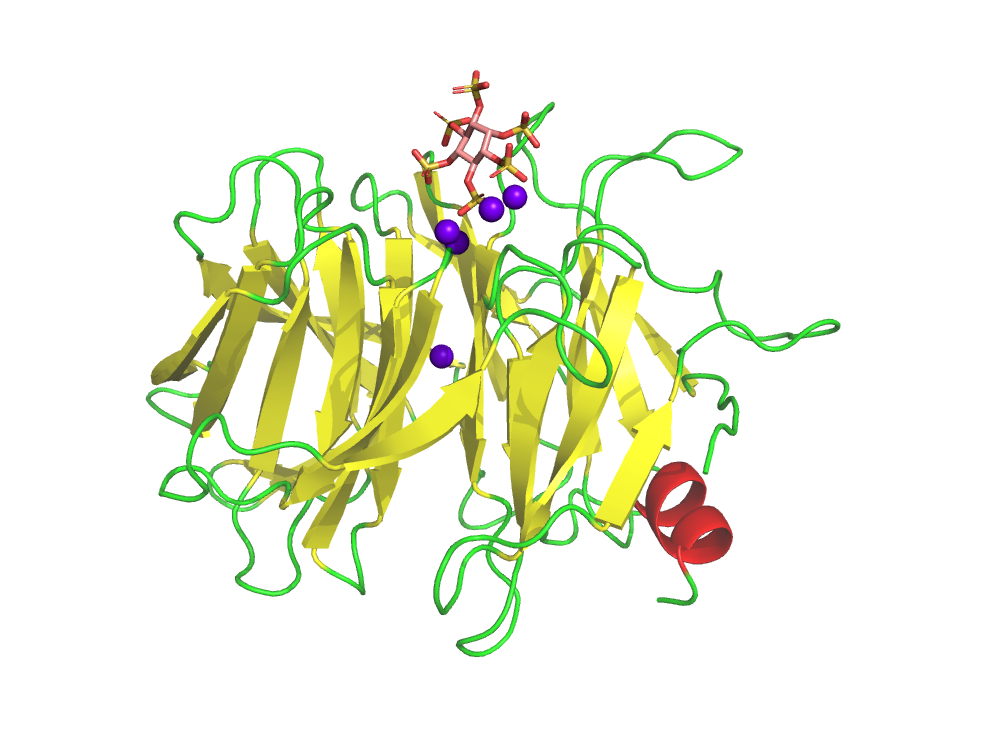

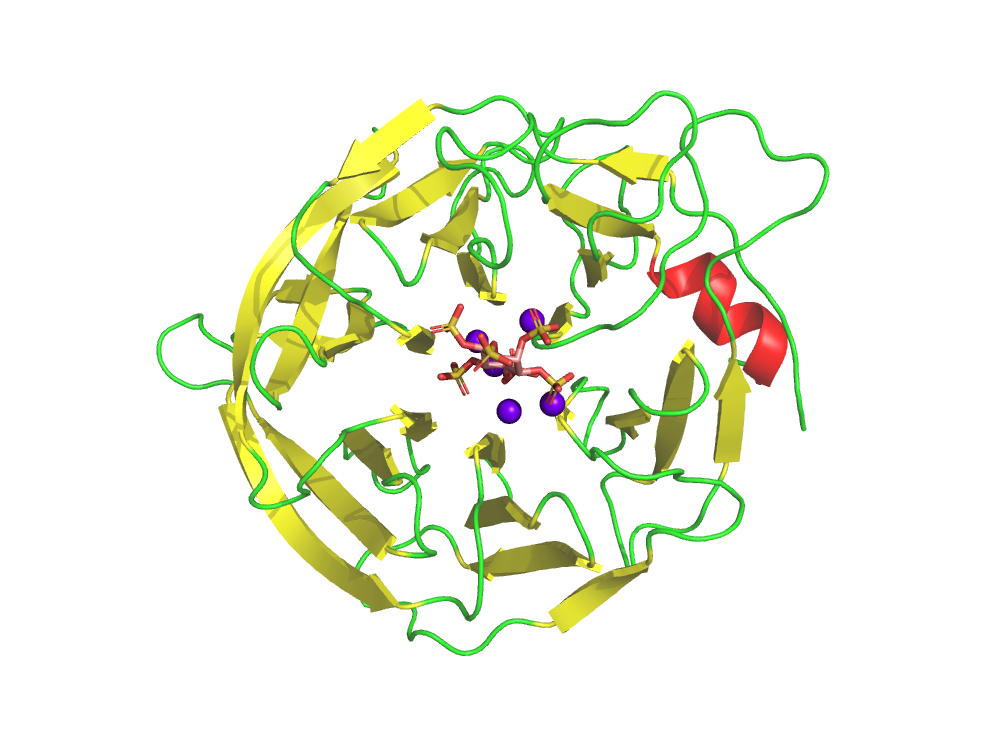

Supplement: Supplementary file 1 [file Table_1.DOCX]
